# Effects of Brewing Temperature and Time on the Quality Attributes and Chemical Profiles of *Cyclocarya paliurus* Tea Infusions

**DOI:** 10.3390/foods15203691

**Published:** 2026-10-16

**Authors:** Yanmeng Fu, Jingyi Zhao, Wenhui Zhou, Jiaxing Ye, Bin Long, Nan Huang, Wei Wang, Xing Tian

**Affiliations:** 1 TCM and Ethnomedicine Innovation & Development International Laboratory, Innovative Material Medical Research Institute, School of Pharmacy, Hunan University of Chinese Medicine, Changsha 410208, China; 2 Department of Food and Drug Engineering, School of Pharmacy, Hunan University of Chinese Medicine, Changsha 410208, China; 3 Hunan Engineering and Technology Research Center for Health Products and Life Science, Changsha 410208, China

**Keywords:** *Cyclocarya paliurus*, cold brewing, hot brewing, sensory quality, volatile compounds, untargeted metabolomics, radical-scavenging capacity

## Abstract

Brewing temperature and duration jointly modulate the sensory perception, functional properties, and chemical composition of herbal tea infusions, yet their integrated effects on *Cyclocarya paliurus* tea remain poorly understood. Here, cold-brewed (4 °C) and hot-brewed (90 °C) *C. paliurus* infusions prepared over a 5 min–2 h time gradient were systematically characterized using a multi-platform analytical strategy. Brewing mode and time produced clearly differentiated quality profiles: hot brewing yielded darker infusions with stronger bitterness and astringency, higher phenolic and flavonoid contents, and greater 2,2-diphenyl-1-picrylhydrazyl (DPPH) and 2,2′-azino-bis(3-ethylbenzothiazoline-6-sulfonic acid) (ABTS) radical-scavenging capacities, whereas cold brewing produced lighter, milder infusions with more gradual time-dependent compositional changes. Volatile and non-volatile profiles were clearly separated between the two brewing modes, and their temporal patterns indicated compound-specific temperature- and time-dependent responses rather than a generalized increase in extraction efficiency at elevated temperature. In total, 61 volatile features and 3666 metabolite features were annotated, from which 50 core differential metabolites were screened; exploratory treatment-level correlation analysis linked tentatively annotated gossypetin 8-glucuronide to radical-scavenging capacity, kaempferol 3-O-arabinoside and sericoside to bitterness-related responses, and epoxypheophorbide a to color variation. These findings clarify how brewing temperature and time jointly shape the quality of *C. paliurus* tea infusions and provide candidate chemical markers to guide the selection of brewing conditions and the development of differentiated *C. paliurus*-based herbal beverages. As a single commercial batch of *C. paliurus* tea was examined, these findings should be interpreted within the context of the tested material and brewing conditions.

## 1. Introduction

*Cyclocarya paliurus* (Batalin) Iljinskaja, a deciduous tree of the Juglandaceae family, is a characteristic edible plant resource native to China whose leaves have traditionally been consumed as a naturally sweet herbal infusion. Its leaves contain diverse phytochemical constituents, including phenolics, flavonoids, polysaccharides, triterpenoids, and saponins, and have attracted increasing interest as raw materials for tea-based and functional beverage products [1,2]. Multiple previous investigations have confirmed that phenolic and flavonoid-rich fractions of *C. paliurus* contribute greatly to its antioxidant capacity, further supporting its potential for value-added beverage development [2]. Nevertheless, the quality and commercial value of tea beverages depend not only on phytochemical composition and potential functional properties, but also on sensory characteristics, including color, aroma, bitterness, astringency, sweetness, and overall taste balance. Therefore, clarifying how brewing conditions simultaneously reshape sensory attributes and functional properties is essential for rational development and the utilization of *C. paliurus* tea products.

Tea infusion quality is a multidimensional outcome resulting from interactions among sensory attributes and chemical constituents. Non-volatile compounds, particularly catechins, flavonoids, phenolic acids, free amino acids, and other soluble metabolites, are closely associated with bitterness, astringency, umami, color development, and antioxidant properties [3,4]. For example, changes in catechins, flavonoid glycosides, and phenolic acids have been associated with alterations in bitterness and astringency, whereas catechins and free amino acids can contribute simultaneously to antioxidant activity and taste characteristics [3,4]. Volatile constituents, including alcohols, aldehydes, esters, ketones, terpenoids, and norisoprenoids, constitute another important chemical basis of tea aroma, although their sensory contribution depends strongly on concentration and odor activity rather than abundance alone [5]. Furthermore, precursor–product transformations between non-volatile substrates and volatile aroma substances add extra complexity to flavor formation during steeping [6]. Accordingly, the overall quality of tea infusions cannot be explained by any single compound or chemical family, but reflects coordinated alterations across both volatile and non-volatile metabolomes.

Brewing temperature and time are major external factors governing mass transfer, compound extraction, and quality development in tea infusions. Higher temperatures generally accelerate the release of water-soluble phytochemicals and can markedly alter the concentration of non-volatile compounds and the sensory attributes of tea infusions [7]. Brewing conditions also affect volatile composition and aroma perception; a study of black tea showed that brewing parameters substantially influenced both phytochemical profiles and sensory quality, while steeping temperature has recently been shown to modify the abundance of key aroma-active compounds in Longjing tea [5,7]. In addition, brewing conditions can affect the distribution and release behavior of volatile compounds into the infusion matrix, meaning that aroma changes cannot be interpreted simply from extraction yield alone [8]. In contrast, cold brewing generally proceeds through slower extraction kinetics and prolonged leaf–water contact and may therefore result in different temporal patterns of phenolics, flavor compounds, and other metabolites [1]. Collectively, these observations imply that brewing-induced quality discrepancies should be interpreted as compound-specific and time-resolved responses instead of a simple outcome of higher extraction efficiency at high temperature.

Although cold-brewed herbal beverages are attracting growing consumer interest, there remain critical knowledge gaps regarding brewing-driven quality divergence in *C. paliurus* tea. A previous study compared discrete brewing treatments without a continuous time-gradient design, and it remains unclear whether quality divergence originates from globally higher extraction efficiency at high temperature or from compound-specific temperature-time dependent responses [1]. Subsequent work demonstrated that volatile and non-volatile constituents jointly determine the flavor characteristics and antioxidant activities of *C. paliurus* infusions [2]. However, these earlier studies were performed at fixed single or limited steeping time points. More importantly, few existing reports integrate descriptive sensory evaluation, electronic tongue taste profiling, volatile fingerprinting and non-volatile metabolomics within one unified experimental framework. As a result, the statistical links between brewing-triggered chemical shifts and multi-dimensional quality traits of *C. paliurus* tea are still poorly characterized; causal relationships between candidate metabolites and quality phenotypes also remain unvalidated. An integrated multi-platform assessment is therefore warranted to further characterize the chemical basis underlying brewing-dependent quality variation in *C. paliurus* tea.

Therefore, the present study systematically investigated *C. paliurus* tea infusions prepared under different cold and hot brewing conditions by integrating color analysis, quantitative descriptive sensory evaluation, electronic tongue analysis, determination of bioactive constituents and in vitro radical-scavenging capacity, HS-SPME-GC-MS, and untargeted metabolomics. We hypothesized that quality differentiation between cold- and hot-brewed *C. paliurus* tea would reflect compound-specific temperature-and time-dependent compositional responses rather than differences in overall extraction efficiency alone. The objectives were to: (1) characterize changes in color, sensory attributes, electronic tongue responses, bioactive constituents and radical-scavenging responses under different brewing conditions; (2) compare the temperature-and time-associated response patterns of volatile and non-volatile chemical constituents; and (3) identify representative differential metabolite features and evaluate their statistical associations with quality-related characteristics. This integrated approach was designed to provide chemical insights into brewing-dependent quality differentiation in *C. paliurus* tea and to inform the selection of brewing conditions for developing herbal beverages with distinct sensory and chemical characteristics.

## 2. Materials and Methods

### 2.1. Materials and Chemicals

Commercial dried *C. paliurus* tea was used as the test material. The tea was processed from fresh young shoots (one bud with one to four leaves) harvested in spring, following the standardized processing procedure specified in the Chinese local standard DB35/T 2087-2022 (Technical requirements for the processing of *Cyclocarya paliurus* tea), comprising withering, rolling, high-temperature fixation (kill-green), re-rolling into uniform strip-shaped leaves, baking at 85–90 °C to a final moisture content of ≤7%, and sorting to remove old leaves, coarse stems, and foreign matter. The tea was supplied by Hunan Yueling Junshan Agriculture and Forestry Technology Co., Ltd., produced in Changde, Hunan Province, China (Batch No. 20250408; finished-product moisture content ≤7%). The tea samples were stored in sealed moisture-proof packaging under dry and dark conditions at room temperature and were analyzed within 3 months of production. Before the experiments, the tea leaves were thoroughly mixed to improve sample homogeneity and randomly selected for infusion preparation.

Purified bottled drinking water (C’estbon; China Resources C’estbon Beverage (China) Co., Ltd., Shenzhen, China) was used for the preparation of all tea infusions. According to the product specification for purified drinking water, the water had a pH of 5.0–7.0 and an electrical conductivity of ≤10 μS/cm (total dissolved solids < 10 mg/L). The same batch of bottled water was used throughout the entire experiment to exclude any influence of water composition on the comparison between brewing conditions.

For the determination of bioactive constituents and in vitro radical-scavenging capacity, Folin–Ciocalteu reagent was purchased from Hefei Bomei Biotechnology Co., Ltd. (Hefei, China). D-(+)-glucose and gallic acid standards were obtained from Chengdu Desite Biotechnology Co., Ltd. (Chengdu, China). Rutin and oleanolic acid standards were purchased from Chengdu Alfa Biotechnology Co., Ltd. (Chengdu, China). Phenol and methanol used for routine colorimetric assays were obtained from Xilong Scientific Co., Ltd. (Shantou, China). Anhydrous aluminum chloride and anhydrous sodium carbonate were purchased from Sinopharm Chemical Reagent Co., Ltd. (Shanghai, China). Concentrated sulfuric acid was purchased from Hunan Huihong Reagent Co., Ltd. (Hunan, China). Sodium acetate buffer and the DPPH radical-scavenging assay kit were obtained from Beijing Labgic Technology Co., Ltd. (Beijing, China), and the ABTS radical-scavenging assay kit was obtained from Jiangsu Aidisheng Biotechnology Co., Ltd. (Yancheng, China).

For untargeted metabolomic analysis, methanol (Cat. No. 1.06007.4008), acetonitrile (Cat. No. 1.00030.4008), and formic acid (Cat. No. 111670) were obtained from Millipore. These reagents were used for metabolite extraction and UHPLC-HRMS/MS analysis. All other chemicals and reagents were of analytical grade unless otherwise specified. Ultrapure water was used for the analytical procedures unless otherwise stated, whereas purified bottled water was used for preparation of the tea infusions.

### 2.2. Preparation of Cold- and Hot-Brewed C. paliurus Tea Infusions

For each brewing treatment, 1.00 ± 0.01 g of dried *C. paliurus* tea leaves was placed in a 100 mL glass beaker and infused with 50 mL of purified bottled water at a tea-to-water ratio of 1:50 (*w*/*v*).

For the hot-brewing treatment, purified water preheated to 90 °C was poured onto the weighed tea leaves, and the beaker was immediately sealed with a double layer of aluminum foil to minimize water evaporation and volatile compound loss during steeping. The infusion system was maintained at 90 °C using a thermostatically controlled water bath throughout the steeping period. For the cold-brewing treatment, purified water pre-cooled to approximately 4 °C was added to the tea leaves, and the beaker was similarly sealed with aluminum foil; the infusion system was maintained in a refrigerated chamber at 4 °C throughout steeping. Water evaporation under each brewing condition was pre-validated in blank experiments without tea leaves: after 2 h of incubation under the corresponding sealed conditions, the volume loss was less than 0.4 mL (<0.8% of the initial volume), indicating that evaporative concentration was negligible within the tested duration.

Both brewing regimes were carried out across five graded time points: 5 min, 10 min, 30 min, 1 h, and 2 h. The obtained infusions were designated C5m, C10m, C30m, C1h, and C2h for the cold-brewing series, and H5m, H10m, H30m, H1h, and H2h for the hot-brewing series, where “C” and “H” denote cold and hot brewing, respectively, and the following alphanumeric suffix corresponds to the applied brewing duration.

Immediately upon reaching the predetermined brewing time, the beaker was transferred to an ice-water bath to rapidly quench the extraction and chemical transformation process. All samples were cooled to 25 °C within 2 min under strictly consistent operating conditions to eliminate differential extraction or compound conversion during the cooling phase. After cooling, the tea infusions were immediately filtered through qualitative filter paper. The tea residues and filter paper were rinsed 2–3 times with small volumes of purified water, and the filtrates were combined. The final volume was adjusted to 50 mL with purified water. For color measurement, electronic tongue analysis, bioactive constituent determination, radical-scavenging assays, and volatile analysis, each brewing treatment was independently prepared in triplicate. For sensory evaluation, one freshly prepared infusion per brewing treatment was assessed by nine trained panelists, as described in Section 2.4.1. For untargeted metabolomics, six independent samples were prepared for each treatment, as described in Section 2.7.

### 2.3. Color Measurement

The color characteristics of cold- and hot-brewed *C. paliurus* tea infusions were measured using a spectrophotometric colorimeter (CS-820N, CHNSpec Technology Co., Ltd., Hangzhou, China). Before measurement, the instrument was calibrated using standard white and black calibration plates.

Tea infusion samples were transferred into transparent sample cells, and color parameters were recorded according to the CIE Lab* color system. The L* value represents lightness, ranging from 0 (black) to 100 (white); a* represents the red (+)–green (−) axis; and b* represents the yellow (+)–blue (−) axis. Each sample was measured three times, and the mean value was used for subsequent statistical analysis.

### 2.4. Sensory Evaluation and Electronic Tongue Analysis

#### 2.4.1. Sensory Evaluation

Sensory profiling of cold- and hot-brewed *C. paliurus* tea infusions was performed by a trained descriptive sensory panel, with reference to the Chinese national standard GB/T 23776-2018 (Methodology of Sensory Evaluation of Tea) with minor modifications. The panel consisted of 9 participants (3 males and 6 females, aged 18–23 years) recruited from undergraduate and postgraduate students at Hunan University of Chinese Medicine. All panelists were non-smokers, without known taste or olfactory disorders. Over a two-week period, all participants received four standardized training sessions to familiarize themselves with tea-related sensory terminology, attribute recognition, and intensity scoring scales before formal testing. Written informed consent was obtained from every panelist prior to participation.

Sensory tests were carried out in individual partitioned booths within a controlled-environment sensory laboratory maintained at 20–25 °C. Evaluations were conducted in the morning (9:00–11:00 a.m.). Tea infusions were freshly prepared as described in Section 2.2 and equilibrated to 25 °C prior to sensory assessment. Each sample was coded with a random three-digit blinding code and presented to panelists in a randomized order to minimize sequence-order bias. Each of the nine panelists evaluated all ten brewing treatments once, yielding nine individual sensory scores per treatment for each attribute. Panelists were requested to avoid consuming strongly flavored foods, spicy dishes, coffee, and alcoholic beverages for 2 h before and throughout the sensory test period.

Quantitative descriptive analysis (QDA) was adopted to quantify sensory attribute intensities. A 10-point intensity scale ranging from 0 (not perceptible) to 10 (extremely intense) was used for scoring. The evaluated sensory attributes included aroma, sweetness, bitterness, sourness, umami, and thickness. Approximately 20 mL of each tea infusion was provided per assessment. Panelists were instructed to take the sample into the oral cavity, ensure full contact with oral mucosal surfaces for several seconds, and then expectorate the liquid after evaluation. Purified bottled water was supplied for palate cleansing between successive samples, and a minimum 5-min resting interval was enforced between different samples to mitigate sensory fatigue and cross-sample carry-over effects.

Individual sensory scores were retained for the assessment of panelist-to-panelist variability. For each brewing treatment, sensory attributes were summarized as the mean ± standard deviation (SD) of the nine panelist scores. Inter-panelist variability was further characterized using coefficients of variation (CVs) and Pearson correlation coefficients between individual panelist profiles and the corresponding panel-mean profiles. An exploratory two-way additive ANOVA with brewing treatment and panelist as factors was conducted to examine systematic differences in scoring levels among panelists. Because each panelist evaluated each treatment only once, the treatment × panelist interaction could not be estimated separately from residual variation. The resulting analyses were interpreted descriptively, and individual panelist scores were not treated as independent infusion-preparation replicates. The summarized results are provided in Supplementary Table S1.

#### 2.4.2. Electronic Tongue Analysis

The taste characteristics of cold- and hot-brewed *C. paliurus* tea infusions were analyzed using an electronic tongue system (TS-5000Z, INSENT Inc., Tokyo, Japan). Before measurement, all samples were equilibrated to room temperature. The system was equipped with artificial lipid-membrane sensors, and the taste attributes used for subsequent analysis included bitterness, astringency, sweetness, umami, bitter aftertaste (Aftertaste-B), astringent aftertaste (Aftertaste-A), and richness. Taste-response indices were calculated from the membrane potentials of the artificial lipid-membrane sensors relative to the reference solution according to the manufacturer’s algorithm.

Prior to sample analysis, the operational status of each sensor was validated according to the manufacturer’s specifications. Aliquots of tea infusion samples were transferred into dedicated sample cups, and taste signal acquisition was performed following the standard operating procedure provided by the manufacturer. After each measurement, the sensor array was thoroughly rinsed with purified water and allowed to re-equilibrate to the baseline potential before analysis of the next sample. Three independently prepared brewing replicates were analyzed for each treatment (*n* = 3). Data acquisition and taste-index calculation were performed using the TS-5000Z system analysis software. Complete electronic tongue response values, including the full instrument outputs, are provided in Supplementary Table S2.

### 2.5. Determination of Bioactive Constituents and Radical-Scavenging Capacity

The contents of total phenolic content (TPC), total flavonoid content (TFC), total polysaccharide content (TP), and total saponin content (TSC) in *C. paliurus* tea infusions were determined using colorimetric assays. Tea infusions were prepared as described in Section 2.2 and centrifuged at 4500 rpm for 5 min before analysis.

All colorimetric reactions were performed in glass tubes. After completion of the reactions, 200 μL of each reaction mixture was transferred into a 96-well microplate, and absorbance was measured using a Spark Multimode Microplate Reader (Tecan Group Ltd., Männedorf, Switzerland). Unless otherwise specified, measurements were performed in triplicate.

#### 2.5.1. Total Phenolic Content

TPC was determined using the Folin–Ciocalteu colorimetric method with slight modifications according to Xiao et al. [9]. Briefly, 1.0 mL of tea infusion was mixed with 3.0 mL of Folin–Ciocalteu reagent diluted 10-fold with distilled water. After standing for 30 s, 6.0 mL of 12% sodium carbonate solution was added, and the mixture was diluted to 25 mL with distilled water. The reaction mixture was incubated at 20 °C in the dark for 2 h, and absorbance was measured at 760 nm. Gallic acid was used as the calibration standard, and the results were expressed as milligrams of gallic acid equivalents per gram of dry tea leaves (mg GAE/g DW).

#### 2.5.2. Total Flavonoid Content

TFC was determined using an aluminum chloride colorimetric method with slight modifications according to Yang et al. [10]. Briefly, 2.0 mL of tea infusion was mixed with 6.4 mL of 1.5% AlCl_3_ solution and 3.2 mL of sodium acetate buffer (0.1 M, pH 5.5). The mixture was adjusted to 20 mL with 50% ethanol and incubated at 25 °C in the dark for 30 min. Absorbance was measured at 415 nm. Rutin was used as the calibration standard, and the results were expressed as milligrams of rutin equivalents per gram of dry tea leaves (mg RE/g DW).

#### 2.5.3. Total Polysaccharide Content

TP was determined using the phenol–sulfuric acid method with slight modifications according to Wu et al. [11]. Briefly, 1.0 mL of tea infusion was mixed with 1.0 mL of 5% phenol solution, followed by the rapid addition of 5.0 mL of concentrated sulfuric acid. The mixture was incubated at 40 °C for 30 min and cooled to room temperature. Absorbance was measured at 490 nm. Glucose was used as the calibration standard, and the results were expressed as milligrams of glucose equivalents per gram of dry tea leaves (mg GE/g DW).

#### 2.5.4. Total Saponin Content

TSC was determined using a vanillin–sulfuric acid colorimetric method with slight modifications according to de Aguiar et al. [12]. Briefly, 100 μL of tea infusion was mixed with 250 μL of 8% vanillin ethanol solution and 2.5 mL of 60% sulfuric acid. The mixture was vortexed for 10 s, incubated at 70 °C for 10 min, and immediately cooled in an ice bath for 5 min. Absorbance was measured at 544 nm. Oleanolic acid was used as the calibration standard, and the results were expressed as milligrams of oleanolic acid equivalents per gram of dry tea leaves (mg OAE/g DW).

#### 2.5.5. In Vitro Radical-Scavenging Assays (DPPH and ABTS)

##### DPPH Radical-Scavenging Capacity

The 2,2-diphenyl-1-picrylhydrazyl (DPPH) radical-scavenging capacity was determined using the method of Brand-Williams et al. with slight modifications [13]. Appropriate dilution factors for the tea infusions were determined in preliminary experiments, and all samples were equilibrated to 25 °C before analysis. To correct for interference from the intrinsic color of the tea infusions, four reaction systems were prepared: sample reaction, 400 μL of diluted tea infusion and 600 μL of DPPH working solution; sample background, 400 μL of diluted tea infusion and 600 μL of 80% ethanol; control, 400 μL of ethanol–water solution and 600 μL of DPPH working solution; and blank, 400 μL of ethanol–water solution and 600 μL of 80% ethanol. A common dilution factor was subsequently applied to all samples to allow for a direct comparison among brewing treatments.

The mixtures were incubated at 25 °C in the dark for 30 min. After incubation, 200 μL of each reaction mixture was transferred into a 96-well microplate, and absorbance was measured at 517 nm.

$$\mathrm{DPPH}(\%)=\left[1-\frac{\left(A_{s}-A_{b}\right)}{\left(A_{0}-A_{e}\right)}\right]\times 100$$

where *A_s_* is the absorbance of the sample reaction tube, *A_b_* is the absorbance of the sample background tube, *A*_0_ is the absorbance of the control tube, and *A_e_* is the absorbance of the blank tube.

##### ABTS Radical Cation-Scavenging Capacity

The 2,2′-azino-bis(3-ethylbenzothiazoline-6-sulfonic acid) (ABTS) radical cation-scavenging capacity was determined according to Re et al. with slight modifications [14]. The ABTS working solution was diluted with anhydrous ethanol to an appropriate concentration before analysis.

The reaction systems consisted of: sample reaction (*A*_1_), 50 μL of tea infusion and 950 μL of ABTS working solution; sample background (*A*_2_), 50 μL of tea infusion and 950 μL of distilled water; control (*A*_0_), 50 μL of distilled water and 950 μL of ABTS working solution; and blank (*A_b_*), 50 μL of distilled water and 950 μL of anhydrous ethanol.

The mixtures were incubated in the dark for 6 min. Subsequently, 200 μL of each reaction mixture was transferred into a 96-well microplate, and absorbance was measured at 734 nm.

$$\mathrm{ABTS}(\%)=\left[1-\frac{\left(A_{1}-A_{2}\right)}{\left(A_{0}-A_{b}\right)}\right]\times 100$$

where *A*_1_, *A*_2_, *A*_0_, and *A_b_* represent the absorbance values of the sample reaction, sample background, control, and blank tubes, respectively. Complete quantitative data for bioactive constituents and radical-scavenging capacities are provided in Supplementary Tables S3 and S4, respectively.

### 2.6. HS-SPME-GC-MS Analysis of Volatile Compounds

Volatile compounds in cold- and hot-brewed *C. paliurus* tea infusions were analyzed using headspace solid-phase microextraction coupled with gas chromatography-mass spectrometry (HS-SPME-GC-MS). Each brewing treatment was independently prepared and analyzed in triplicate.

For HS-SPME extraction, 5.0 mL of tea infusion was transferred into a 15 mL headspace vial and equilibrated at 60 °C for 10 min. Before use, the 50/30 μm DVB/CAR/PDMS fiber (Supelco, Bellefonte, PA, USA) was conditioned at 250 °C for 30 min according to the manufacturer’s instructions. The preconditioned fiber was then inserted into the glass vial (approximately 1 cm above the sample surface) and exposed to the headspace at 60 °C for 45 min for extraction. Immediately after extraction, the SPME fiber was inserted into the GC injection port and thermally desorbed at 250 °C for 5 min for subsequent GC-MS analysis.

The oven temperature program was as follows: 40 °C for 1 min; increased to 80 °C at 2 °C/min and held for 4 min; increased to 90 °C at 2 °C/min and held for 1 min; increased to 150 °C at 5 °C/min and held for 1 min; increased to 180 °C at 8 °C/min and held for 1 min; and finally increased to 230 °C at 15 °C/min and held for 3 min.

Mass spectrometric analysis was performed in electron ionization mode at 70 eV. The ion source, transfer line, and quadrupole temperatures were set at 230, 250, and 150 °C, respectively. Full-scan data were acquired over an *m*/*z* range of 30–600.

Volatile compounds were tentatively annotated by comparison of their mass spectra with the NIST 23 mass spectral library and by comparison of experimentally determined retention indices (RIs) with reference RI values. Experimental RIs were calculated using a homologous series of n-alkanes analyzed under the same chromatographic conditions. Candidate annotations with |ΔRI| ≤ 30 were retained. Compounds detected in at least two out of the three independent replicates within a brewing treatment were retained for subsequent analysis. Relative content (%) was calculated as the mean peak area of each retained compound divided by the sum of the mean peak areas of all retained compounds within the corresponding brewing treatment and multiplied by 100. RSD (%) was calculated from the available replicate peak-area measurements (*n* = 2 or 3). Missing peaks were treated as not detected and were not replaced by zero. Because authentic standards were not analyzed, all compound assignments were considered tentative, and the peak-area responses were interpreted as relative instrumental responses rather than absolute concentrations. Total ion chromatograms (TICs) of the three independently prepared replicates for each brewing treatment are shown in Supplementary Figure S1. Detailed information on retention time, calculated RI, reference RI, relative content, and RSD for the tentatively annotated volatile compounds is provided in Supplementary Table S5.

### 2.7. Untargeted Metabolomics Analysis

Untargeted metabolomic profiling of non-volatile constituents in cold- and hot-brewed *C. paliurus* tea infusions was performed using ultra-high-performance liquid chromatography coupled with high-resolution tandem mass spectrometry (UHPLC-HRMS/MS).

A total of 60 independent tea infusion samples were analyzed, comprising five cold-brewed treatments (C5m, C10m, C30m, C1h, and C2h) and five hot-brewed treatments (H5m, H10m, H30m, H1h, and H2h), with six independent samples for each treatment (*n* = 6). Quality control (QC) samples were prepared by pooling equal aliquots of all experimental samples. QC samples were used to equilibrate the LC-MS system and monitor analytical reproducibility throughout the analytical sequence. One QC sample was injected after every 15 experimental samples. Analytical stability was evaluated using Hotelling’s T2 statistics together with the clustering behavior of QC samples in PCA (Supplementary Figure S2).

#### 2.7.1. Metabolite Extraction

Tea infusion samples were thawed at 4 °C and thoroughly vortex-mixed. An aliquot of 1.0 mL of each sample was transferred into a centrifuge tube and evaporated to dryness using a vacuum centrifugal concentrator. The dried residue was reconstituted in 1.0 mL of precooled methanol/water (4:1, *v*/*v*), mixed thoroughly, sonicated in an ice bath for 20 min, and incubated at −20 °C for 1 h.

The samples were subsequently centrifuged at 16,000× *g* for 20 min at 4 °C. The supernatants were collected and evaporated to dryness. Before LC-MS/MS analysis, the dried extracts were reconstituted in 400 μL of precooled methanol/water (1:1, *v*/*v*) and centrifuged at 20,000× *g* for 15 min at 4 °C. The resulting supernatants were used for instrumental analysis.

#### 2.7.2. UHPLC Conditions

Chromatographic separation was performed using a Shimadzu Nexera X2 LC-30AD UHPLC system (Shimadzu, Kyoto, Japan) equipped with an ACQUITY UPLC HSS T3 column (2.1 × 100 mm, 1.8 μm; Waters, Milford, MA, USA). The column temperature was maintained at 40 °C, the flow rate was 0.3 mL/min, and the injection volume was 16 μL. Samples were maintained at 4 °C in the autosampler throughout the analytical sequence.

The mobile phases consisted of 0.1% formic acid in water (solvent A) and 0.1% formic acid in acetonitrile (solvent B). The gradient elution program was as follows: 0.01–1.00 min, 2% B; 1.00–5.00 min, 2–48% B; 5.00–7.00 min, 48–80% B; 7.00–11.00 min, 80–100% B; 11.00–13.00 min, 100% B; 13.00–13.01 min, 100–2% B; and 13.01–15.00 min, 2% B. One QC sample was injected after every 15 experimental samples to monitor system stability and data reproducibility.

#### 2.7.3. Mass Spectrometry Conditions

Mass spectrometric analysis was performed using a Q Exactive Plus high-resolution mass spectrometer (Thermo Scientific, Waltham, MA, USA) equipped with a heated electrospray ionization (HESI) source. Data were acquired in both positive and negative ionization modes. The spray voltages were 3.8 kV in positive mode and 3.2 kV in negative mode. The capillary temperature was 320 °C, the probe heater temperature was 350 °C, the sheath gas setting was 30, the auxiliary gas setting was 5, and the S-lens RF level was 50.

The MS acquisition time was 15 min. Full-scan MS data were acquired over an *m*/*z* range of 75–1050 at a resolution of 70,000 at *m*/*z* 200, with an automatic gain control (AGC) target of 3 × 10^6^ and a maximum injection time of 100 ms. Data-dependent acquisition (DDA) was performed by selecting the 10 most intense precursor ions following each full scan for MS/MS acquisition. MS/MS spectra were acquired at a resolution of 17,500 at *m*/*z* 200, with an AGC target of 1 × 10^5^ and a maximum injection time of 50 ms. Higher-energy collisional dissociation (HCD) was used with an isolation window of 2 *m*/*z* and stepped normalized collision energies of 20, 30, and 40.

#### 2.7.4. Data Processing and Metabolite Annotation

Raw UHPLC-HRMS/MS data were processed using MS-DIAL version 4.9 for peak detection, alignment, retention-time correction, and peak-area extraction. Metabolite features were tentatively annotated by matching accurate precursor masses and MS/MS fragmentation spectra against the applied in-house plant metabolite spectral database provided by Shanghai Bioprofile Biotechnology Co., Ltd. (Shanghai, China) using mass tolerances of <20 ppm for precursor ions and <0.01 Da for MS/MS fragments. Because authentic reference standards were not analyzed, the resulting metabolite assignments were regarded as tentative database-based annotations rather than definitive compound identifications. Batch effects were corrected using the statTarget package. Ion features with >50% missing values within a group were excluded. Positive- and negative-ion datasets were separately normalized by total peak area and subsequently concatenated into a combined feature matrix for downstream multivariate and differential analyses.

### 2.8. Statistical Analysis

All data are expressed as mean ± standard deviation (SD) unless otherwise specified. For color measurement, electronic tongue analysis, bioactive constituent quantification, radical-scavenging assays, and HS-SPME-GC-MS analysis, three independently prepared brewing replicates were included for each treatment (*n* = 3). Sensory attributes were summarized using the individual scores of nine panelists who each evaluated the ten brewing treatments once. For untargeted metabolomics analysis, six independent biological replicates were analyzed per brewing treatment (*n* = 6).

Two-way ANOVA was used to evaluate the main effects of brewing mode, brewing time, and their interaction on color parameters, electronic tongue responses, bioactive constituent contents, and radical-scavenging capacities. When significant effects were detected, Duncan’s multiple-range test was used for post hoc comparisons.

For the GC-MS dataset, the effects of brewing temperature, time, and their interaction on the number of detected volatile features were evaluated using a Poisson generalized linear model (GLM) with a log link function, with both factors treated as categorical variables. Ordinary two-way ANOVA was additionally performed as a sensitivity analysis.

Pearson correlation analysis was performed to evaluate pairwise associations among selected sensory attributes, electronic tongue responses, bioactive constituents, radical-scavenging indices, volatile compounds, and metabolites, as appropriate. Correlations were interpreted as statistical associations rather than evidence of causal relationships. For the metabolite–quality correlation analysis, *p* values were adjusted for multiple testing using the Benjamini–Hochberg false discovery rate (FDR) procedure, and adjusted *p* values are reported as q values.

Principal component analysis (PCA) and orthogonal partial least-squares discriminant analysis (OPLS-DA) were performed for the GC-MS and untargeted metabolomics datasets using SIMCA 14.1 (Umetrics, Umeå, Sweden). OPLS-DA models were evaluated using R^2^X, R^2^Y, and Q^2^ values and validated using 200-permutation tests. Variable importance in projection (VIP) values were used as one criterion for screening candidate differential variables.

For untargeted metabolomics, differential features were screened across five matched-time cold–hot comparisons using VIP > 1, FDR < 0.05, and |log_2_FC| > 1, followed by the selection of 50 representative features. Additionally, full-factorial two-way ANOVA was performed on 3666 annotated features to assess the effects of brewing temperature, time, and their interaction using log_10_(x + 1)-transformed responses. *p*-values were adjusted using the Benjamini–Hochberg procedure separately for each effect (q < 0.05), and interaction effects were further evaluated using HC3 heteroscedasticity-robust tests. Detailed results for the 50 representative features are provided in Supplementary Tables S6 and S7. Hierarchical clustering and heatmap visualization were performed using TBtools v1.132 (Guangzhou, China).

Statistical analyses and graphical presentations were conducted using SPSS 26.0 (IBM Corp., Armonk, NY, USA), Origin 2021 (OriginLab Corporation, Northampton, MA, USA), GraphPad Prism 9.0 (GraphPad Software, San Diego, CA, USA), SIMCA 14.1, and TBtools, as appropriate.

## 3. Results

### 3.1. Color, Sensory Characteristics, and Electronic Tongue Responses of Cold- and Hot-Brewed C. paliurus Tea Infusions

As expected for the tea infusions, the visual color deepened progressively with increasing brewing time under both brewing modes (Figure 1A), consistent with previous reports on conventionally brewed and cold-brewed teas [15,16,17]. This anticipated behavior confirms that the brewing system performed as intended and provides a reference frame for the quantitative comparisons below. Cold-brewed samples generally maintained higher L* values and lower a* and b* values, resulting in a lighter yellow-green appearance, whereas hot-brewed samples showed progressively lower L* values and higher a* and b* values with prolonged brewing, indicating more pronounced color development.

Descriptive sensory evaluation revealed differences in the sensory profiles of cold- and hot-brewed *C. paliurus* tea infusions (Figure 1B, upper). Across the ten brewing treatments, the nine trained panelists provided scores for aroma, sweetness, bitterness, sourness, umami, and thickness. The resulting panel-mean profiles illustrated brewing-dependent differences in perceived sensory attributes. These patterns were interpreted descriptively because each treatment was evaluated once by each panelist, without independent sensory replicate preparations. Inter-panelist variability and scoring consistency are summarized in Supplementary Table S1.

Electronic tongue analysis yielded taste response patterns broadly consistent with the sensory evaluation results (Figure 1B, lower; Figure 1C), while the complete response values for all measured taste attributes are provided in Supplementary Table S2. The radar profiles of the electronic tongue responses showed that hot-brewed infusions generated stronger signals for bitterness, astringency, bitter aftertaste (Aftertaste-B), and astringent aftertaste (Aftertaste-A). In contrast, cold-brewed samples exhibited higher sweetness and umami response values. Quantitative comparison of representative taste indices (Figure 1C) further showed that the hot-brewed samples exhibited higher bitterness, astringency, and aftertaste responses, whereas cold-brewed samples maintained relatively higher sweetness responses over the 2-h brewing period.

The electronic tongue response patterns can be rationalized in physicochemical terms. The artificial lipid-membrane sensors of the electronic tongue respond to taste-active compounds mainly according to their adsorption behavior at the lipid membrane, which is governed by compound hydrophobicity and charge. The stronger bitterness, astringency, and aftertaste signals of the hot-brewed infusions are therefore consistent with the temperature-accelerated solubilization of polyphenolic and saponin constituents (Figure 2A), which interact with the lipid membranes. Conversely, the relatively higher sweetness and umami responses of the cold-brewed infusions likely reflect two effects: (i) soluble sugars and sweet or umami free amino acids are readily extracted even at 4 °C, whereas the extraction of bitter and astringent polyphenols is markedly retarded at low temperature, shifting the taste balance; and (ii) the lower levels of bitter and astringent compounds reduce masking of the sweetness response. This interpretation is consistent with the metabolite-level associations described in Section 3.5 and with previous electronic tongue–metabolomics studies of *C. paliurus* [18].

### 3.2. Bioactive Constituents and Radical-Scavenging Capacity of Cold- and Hot-Brewed C. paliurus Tea Infusions

Brewing temperature and time markedly affected the levels of total phenolics (TPC), total flavonoids (TFC), total polysaccharides (TP), and total saponins (TSC) in *C. paliurus* tea infusions (Figure 2A). Overall, hot-brewed samples contained higher levels of several bioactive constituents than the corresponding cold-brewed samples. The complete quantitative data for TPC, TFC, TP, and TSC, expressed as mean ± SD, are provided in Supplementary Table S3.

During cold brewing, TPC and TP generally increased with brewing time and reached their highest levels at 2 h. In contrast, both TPC and TP showed a more pronounced increase under hot-brewing conditions, likewise reaching their highest levels at 2 h. TFC showed a similar temporal pattern, with a more pronounced increase during hot brewing. TSC showed relatively smaller variations among treatments. Temperature-dependent differences in the extraction of phenolic and flavonoid constituents have also been observed in tea infusions and *C. paliurus* extracts [19,20].

DPPH and ABTS radical-scavenging capacities differed significantly between the two brewing modes (Figure 2B,C). Two-way ANOVA revealed that brewing mode, brewing time, and their interaction all exerted highly significant effects on both DPPH and ABTS radical-scavenging capacities (*p* < 0.001), with hot-brewed infusions consistently exhibiting stronger scavenging capacity than cold-brewed counterparts at every matched brewing time point. The complete DPPH and ABTS radical-scavenging data, expressed as mean ± SD, are provided in Supplementary Table S4.

For DPPH radical-scavenging capacity, cold-brewed samples increased from 5 min to 1 h and declined at 2 h, whereas hot-brewed samples increased progressively with brewing time and reached the highest response at 2 h. A broadly similar temporal pattern was observed for ABTS radical-scavenging capacity. Previous studies have likewise reported close relationships among phenolic constituents, flavonoids, and antioxidant responses in *C. paliurus* aqueous extracts [20,21].

Pearson correlation analysis further revealed associations among electronic tongue responses and radical-scavenging indices (Figure 2D). Bitterness, astringency, and aftertaste-related responses were generally positively correlated with several bioactive constituent and radical-scavenging variables, whereas sweetness showed weaker or negative correlations with most of these parameters. Comparable associations between non-volatile constituents and sweetness- or bitterness-related characteristics have also been reported in integrated electronic tongue–metabolomics studies of *C. paliurus* leaves [18].

### 3.3. Volatile Profiles of Cold- and Hot-Brewed C. paliurus Tea Infusions

#### 3.3.1. Overall Variation in Volatile Profiles

A total of 61 volatile features were retained across the ten brewing treatments and tentatively annotated based on mass-spectral library matching and retention-index consistency (Supplementary Table S5). Representative total ion chromatograms (TICs) of the three independently prepared replicates for each brewing condition are shown in Supplementary Figure S1. These features were mainly assigned to alcohols/phenols, aldehydes, esters, hydrocarbons, ketones, nitrogen-containing compounds, and other compound classes (Figure 3A). Similar volatile classes have previously been reported in *C. paliurus* tea and related herbal extracts [22,23].

The number of detected volatile features and their relative class distributions differed among brewing conditions. Under cold-brewing conditions, the number of detected features increased from 8 in C5m to 15 in C2h. In contrast, 21 and 23 volatile features were detected in H5m and H10m, respectively, indicating a more pronounced early-stage HS-SPME-GC-MS response under hot-brewing conditions. A Poisson GLM revealed a significant temperature × time interaction in replicate-level volatile feature counts (Wald χ^2^ = 12.641, df = 4, *p* = 0.013), which was supported by ordinary two-way ANOVA (F(4, 20) = 6.349, *p* = 0.0018). As shown in Figure S3, the number of detected features generally increased with cold-brewing duration, whereas hot-brewed samples exhibited higher feature counts at earlier time points followed by a decrease during prolonged brewing. These findings indicate distinct temporal detection patterns between the two brewing modes.

The relative peak-area proportions of individual volatile classes also varied with brewing time. Hydrocarbons accounted for 46.36% and 38.28% of the total normalized volatile signal in C5m and C10m, respectively. With prolonged cold brewing, aldehyde-associated signals represented 52.17% of the total normalized peak area in C30m and 54.17% in C2h. Under hot-brewing conditions, aldehydes represented 53.71%, 50.34%, and 40.80% of the total normalized peak area in H5m, H30m, and H2h, respectively, whereas alcohol/phenol-associated signals represented 35.05% in H10m. Because HS-SPME responses are influenced by compound volatility, matrix effects, fiber affinity, and gas–liquid partitioning, these relative peak-area proportions should not be interpreted as absolute concentrations of individual VOCs in the tea infusions. Compound-specific retention times, calculated and reference RIs, relative contents, and RSD values are provided in Supplementary Table S5.

PCA based on the normalized GC-MS responses revealed treatment-dependent variation in volatile profiles (Figure 3B). PC1 and PC2 explained 18.2% and 13.7% of the total variance, respectively, accounting for a combined 31.9%, while the three-component PCA model explained 42.9% of the variance [R^2^X(cum) = 0.429]. The score plot showed that cold-brewed samples were generally clustered within a relatively narrow region, whereas hot-brewed samples exhibited greater dispersion, particularly for the H5m and H10m treatments. However, the two brewing modes were not completely separated, and brewing duration did not produce a consistent monotonic shift along either principal component. These patterns indicate treatment-specific variation in volatile profiles rather than a simple separation driven exclusively by brewing temperature or duration. Time-dependent changes in volatile response profiles during tea brewing have also been reported using real-time aroma-profiling approaches [24].

OPLS-DA further differentiated the two brewing modes, with R^2^X(cum), R^2^Y(cum), and Q^2^(cum) values of 0.756, 0.858, and 0.602, respectively (Figure 3C). A 200-permutation test yielded a negative Q^2^ intercept (−0.404). Based on the OPLS-DA model, 28 candidate discriminant volatile features with VIP > 1 were screened, and the top 20 features ranked by VIP were subjected to hierarchical clustering analysis (Figure 3D). These features exhibited distinct temperature- and time-dependent normalized response patterns.

#### 3.3.2. Representative Discriminant Volatile Features

Seven representative volatile features tentatively annotated as benzaldehyde, 2-methylbutanal, heptanal, 4-hexen-1-ol, fenchyl acetate, β-phellandrene, and 3,6,6-trimethylcyclohex-2-enone were selected from these candidate discriminant features based on differences between brewing modes, detection frequency, temporal response patterns, and chemical-class representativeness.

Pearson correlation analysis revealed significant statistical associations between several representative volatile features and sensory or electronic-tongue variables (Figure 3E). In particular, features tentatively assigned as fenchyl acetate, heptanal, and 3,6,6-trimethylcyclohex-2-enone were significantly correlated with several bitterness-, astringency-, or electronic-tongue-related parameters. These correlations indicate coordinated variation among brewing treatments but do not demonstrate direct sensory effects of the corresponding compounds.

Benzaldehyde, 2-methylbutanal, and heptanal have previously been reported among volatile constituents associated with characteristic aroma attributes in tea systems [25,26]. However, the present study did not determine absolute concentrations, odor thresholds, odor activity values, or GC–olfactometry responses. Therefore, the observed differences in normalized GC-MS responses should be interpreted as differences in volatile chemical profiles rather than direct evidence of the aroma contribution of individual compounds.

### 3.4. Untargeted Metabolomic Profiles and Differential Metabolites of Cold- and Hot-Brewed C. paliurus Tea Infusions

#### 3.4.1. Overall Metabolic Profiles

Untargeted metabolomics yielded 3666 database-annotated metabolite features across the cold- and hot-brewed *C. paliurus* tea infusions. Among these, 2557 metabolites were assigned to defined superclass categories, whereas 1109 metabolites were classified as unclassified and excluded from the superclass distribution shown in Figure 4A.

Quality-control evaluation indicated satisfactory analytical stability. The Hotelling’s T2 plot showed that the overall sample distribution remained within the predefined confidence boundaries, while QC samples clustered closely in the PCA score plot (Supplementary Figure S2), supporting satisfactory reproducibility of the UHPLC-HRMS/MS analytical platform. The classified metabolites were mainly assigned to lipids and lipid-like molecules, organoheterocyclic compounds, organic acids and derivatives, phenylpropanoids and polyketides, organic oxygen compounds, and benzenoids. The broad metabolite distribution was generally consistent with previous reports showing that *C. paliurus* leaves contain diverse flavonoid-, phenolic-, lipid-, organic acid-, and triterpenoid-related metabolites [27].

PCA based on the 3666 tentatively annotated metabolite features showed clear variation among brewing treatments (Figure 4B). PC1 and PC2 explained 49.04% and 14.25% of the total variance, respectively, corresponding to a cumulative explained variance of 63.29%.

OPLS-DA further differentiated cold- and hot-brewed samples (Figure 4C), with R^2^X, R^2^Y, and Q^2^ values of 0.784, 0.985, and 0.877, respectively. A 200-permutation test yielded a negative Q^2^ intercept. These multivariate results showed distinct overall non-volatile metabolite profiles between the two brewing modes.

#### 3.4.2. Differential Metabolite Responses

Paired comparisons between cold- and hot-brewed samples at matched brewing times revealed pronounced time-dependent differences in the relative abundance of metabolite features (Figure 4D). At 5 min, 1649 metabolite features showed higher relative abundance in hot-brewed samples than in the corresponding cold-brewed samples, and this number progressively decreased to 983 at 2 h. In contrast, the number of metabolite features showing higher relative abundance in the cold-brewed samples increased from 491 at 5 min to 854 at 2 h. Previous untargeted metabolomics work has similarly demonstrated substantial differences among *C. paliurus* infusions prepared using different brewing procedures [28].

To further assess the combined effects of brewing temperature and time, full-factorial two-way ANOVA was performed on the 3666 annotated metabolite features. After Benjamini–Hochberg FDR correction (q < 0.05), 2926 features (79.8%) exhibited significant temperature × time interactions. Among the 50 representative differential metabolite features displayed in Figure 4E, 47 showed significant interactions, of which 46 were also supported by HC3 heteroscedasticity-robust analysis. Overall, 2837 out of the 2926 interaction-significant features remained significant under HC3 analysis, supporting widespread temperature-dependent temporal response patterns. Detailed statistical results for the 50 representative features are provided in Supplementary Table S7.

Representative differential metabolite features were further screened from the database-annotated dataset using VIP > 1, FDR < 0.05, |log_2_FC| > 1, together with the predefined consistency criterion across matched-time comparisons. A total of 50 core differential metabolite features were retained and subjected to hierarchical clustering analysis (Figure 4E). Detailed classification, tentative annotation, statistical information, and brewing-response patterns of these 50 features are provided in Supplementary Table S6.

Among the 50 core differential metabolite features, 24 (48%) were classified as lipids and lipid-like molecules, 11 (22%) as phenylpropanoids and polyketides, 9 (18%) as organoheterocyclic compounds, 3 (6%) as organic acids and derivatives, 2 (4%) as benzenoids, and 1 (2%) as an organic nitrogen compound. Several tentatively annotated flavonoid-related metabolites, including kaempferol 3-O-arabinoside and gossypetin 8-glucuronide, were assigned to the phenylpropanoid and polyketide category, whereas tentatively annotated organoheterocyclic compounds included caffeine, lancerin, methyl pheophorbide a, and epoxypheophorbide a. Because these annotations were primarily based on accurate-mass and MS/MS spectral matching, metabolites not confirmed using authentic standards were regarded as tentative annotations rather than definitive identifications [29,30].

### 3.5. Associations Between Core Differential Metabolites and Quality Characteristics

Pearson correlation analysis was performed between the 50 core differential metabolite features and 22 quality-related parameters, including color parameters, sensory attributes, electronic tongue responses, bioactive constituent contents, and radical-scavenging capacities (Figure 5). Correlations were calculated using the treatment-level mean values of the ten brewing conditions (*n* = 10), rather than individual biological replicates. Given the limited number of treatment-level observations, these correlations were considered exploratory and were interpreted as statistical associations rather than evidence of causal relationships.

Several metabolite features showed strong associations with radical-scavenging-related parameters. Gossypetin 8-glucuronide was positively correlated with DPPH (r = 0.876, q = 0.005) and ABTS (r = 0.950, q = 0.002) radical-scavenging capacities. Kaempferol 3-O-arabinoside was positively correlated with ABTS radical-scavenging capacity (r = 0.894, q = 0.003), whereas sericoside was positively correlated with DPPH (r = 0.896, q = 0.003) and ABTS (r = 0.966, q = 0.002). Strong associations were also observed between selected metabolite features and bitterness-related responses. Kaempferol 3-O-arabinoside was positively correlated with sensory bitterness (r = 0.945, q = 0.002) and electronic tongue bitterness response (r = 0.942, q = 0.002). Sericoside showed similar positive correlations with sensory bitterness (r = 0.920, q = 0.002) and electronic tongue bitterness response (r = 0.928, q = 0.002). In contrast, caffeine was not significantly correlated with either sensory bitterness (r = 0.293, q = 0.508) or electronic tongue bitterness response (r = 0.429, q = 0.306).

Several metabolite features were also associated with infusion color. Epoxypheophorbide a showed a strong negative correlation with L* (r = −0.958, q = 0.002) and positive trends with a* and b*, indicating a close statistical association with overall color variation. Overall, the treatment-level correlation analysis revealed distinct statistical associations between brewing-dependent metabolite variation and multiple quality dimensions. Gossypetin 8-glucuronide was mainly associated with antioxidant-related parameters, kaempferol 3-O-arabinoside and sericoside with bitterness-related responses, and epoxypheophorbide a with color variation.

## 4. Discussion

### 4.1. Sensory and Bio-Active Property Differentiation Driven by Brewing Conditions

Brewing temperature and steeping duration jointly shaped the sensory profiles and bioactive constituent levels of *C. paliurus* tea infusions. Hot-brewed infusions exhibited deeper color, stronger bitterness and astringency, and higher contents of phenolics, flavonoids, and polysaccharides, together with greater in vitro radical-scavenging capacity, than their cold-brewed counterparts at equivalent time points (Figure 1 and Figure 2). These outcomes are consistent with temperature-accelerated mass transfer, in which elevated temperature improves solvent penetration into leaf tissues and promotes the solubilization of water-soluble phytochemicals [19]. Importantly, however, the temporal trajectories of these indices differed between the two brewing modes rather than simply scaling with the overall amount extracted: under cold brewing, TPC and TP generally increased throughout the 2-h steeping period, whereas DPPH and ABTS radical-scavenging capacities increased up to 1 h and subsequently declined at 2 h, whereas under hot brewing they accumulated continuously throughout the 2 h period (Figure 2). Similar non-linear temporal trends during cold steeping have been reported for green tea, in which prolonged brewing did not necessarily lead to further increases in soluble phenolic contents [16]. Several processes including shifts in soluble insoluble partitioning, oxidation, or matrix association could potentially contribute to the slight decline observed after prolonged cold steeping; however, these possibilities were not directly examined in the present study and warrant targeted investigation.

Notably, TFC increased continuously throughout the 2 h hot-brewing period without an apparent plateau. This pattern may reflect enhanced extraction of flavonoid-related constituents, release of bound compounds, matrix effects, or changes in compound availability under elevated brewing temperature, rather than necessarily indicating the thermal stability of individual flavonoid glycosides. The present data cannot distinguish among these processes, and compound-level quantification with authentic standards would be required to clarify the underlying mechanism.

### 4.2. Temperature- and Time-Dependent Remodeling of Volatile and Non-Volatile Metabolomes

Untargeted metabolomics and volatile profiling further revealed that brewing temperature and time were associated with distinct changes in both non-volatile and volatile chemical profiles, with responses differing among compound classes rather than showing a uniform increase or decrease across the entire metabolome. Interestingly, lipid- and lipid-like molecules accounted for the largest proportion (48%) of the selected 50 core differential metabolite features. This distribution indicates that brewing-dependent differentiation in *C. paliurus* tea was not restricted to phenolic-related constituents but involved multiple chemical classes [19,28]. This finding suggests that brewing-dependent chemical differentiation in *C. paliurus* extends beyond the phenolic fraction and involves multiple classes of metabolites. The observed differences in lipid- and lipid-like metabolite features may reflect differences in release, transformation, matrix association, or analytical response under different brewing conditions; however, the present data do not allow these possible processes to be distinguished. Because most metabolite annotations were based on accurate-mass and MS/MS spectral matching without confirmation using authentic standards, the corresponding compound assignments should be regarded as tentative rather than definitive.

The temporal distribution of differential metabolite features further indicated that the chemical distinction between cold and hot brewing was dynamic rather than fixed. At the early stage of brewing, a larger number of metabolite features showed relatively higher abundance under hot-brewing conditions, whereas this number progressively decreased with prolonged steeping. In contrast, the number of metabolite features showing relatively higher abundance under cold-brewing conditions increased over time. Similar brewing-dependent metabolic differences have previously been reported for *C. paliurus* infusions prepared using different procedures [1]. These patterns are consistent with different temperature- and time-dependent compositional responses between the two brewing modes, with hot brewing showing a more pronounced early-stage response and cold brewing exhibiting more gradual changes during prolonged steeping. However, because the present study did not directly quantify extraction kinetics, transformation rates, or equilibrium behavior, these temporal trends should be interpreted as relative compositional patterns rather than direct evidence of specific mass-transfer or reaction mechanisms.

Volatile profiling showed a similar temperature- and time-dependent pattern. Hot-brewed samples exhibited a more pronounced early-stage HS-SPME-GC-MS response, whereas the number of detectable volatile features increased progressively during cold brewing. Because HS-SPME-GC-MS peak areas are influenced not only by analyte levels but also by compound volatility, matrix effects, gas–liquid partitioning, and fiber affinity, the normalized peak-area responses obtained here should be regarded as semi-quantitative instrumental signals rather than absolute concentrations in the tea infusions. Accordingly, the observed differences indicate distinct volatile response patterns between brewing modes rather than differences in total volatile yield.

The volatile compounds in the present study were tentatively annotated using mass-spectral library matching together with retention-index consistency, with candidate annotations retained when the absolute difference between the experimental and reference RI values was within 30 RI units. This RI-based screening increased the confidence of the tentative annotations; nevertheless, authentic reference standards were not analyzed, and the compound assignments therefore cannot be regarded as confirmed identifications. Benzaldehyde, 2-methylbutanal, and heptanal have previously been reported in association with characteristic aroma attributes in other tea systems [25,26]. However, these literature-reported odor characteristics provide only contextual information for the present annotations. Because absolute concentrations, odor thresholds, odor activity values, and GC–olfactometry responses were not determined, the actual sensory contributions of these individual volatile compounds to the aroma of *C. paliurus* tea remain unverified.

### 4.3. Metabolite Features Associated with Quality Traits

Exploratory correlation analysis identified several tentatively annotated metabolite features that were statistically associated with specific quality dimensions. These associations were calculated using treatment-level means and therefore should not be interpreted as evidence of direct causal relationships. Gossypetin 8-glucuronide showed strong positive associations with both DPPH and ABTS radical-scavenging capacities. Previous chemical studies have reported appreciable radical-scavenging and reducing properties for gossypetin-derived glycosides and glucuronides [31], providing contextual support for the observed association. Kaempferol 3-O-arabinoside and sericoside were strongly associated with both sensory and electronic-tongue bitterness responses. Kaempferol glycosides have previously been linked to bitter and astringent perception in plant-derived food matrices [32], whereas saponin-related compounds have been associated with bitterness and astringency through interactions with oral sensory systems [33]. These literature reports support the plausibility of the observed associations but do not establish these compounds as direct determinants of bitterness in *C. paliurus* tea.

The absence of a significant correlation between caffeine and bitterness is noteworthy. Although caffeine is a well-established bitter compound in conventional Camellia sinensis tea [34], the present result indicates that variation in the tentatively annotated caffeine feature did not closely track the brewing-dependent bitterness pattern of *C. paliurus* tea under the tested conditions. Because caffeine was not quantitatively confirmed using an authentic standard, its absolute contribution to bitterness cannot be determined from the present dataset.

Epoxypheophorbide a showed a strong negative association with L* and positive trends with a* and b*. Pheophorbide-related compounds are products of chlorophyll degradation, and thermal processing has previously been associated with chlorophyll transformation in tea materials [35,36]. Accordingly, the observed correlation may reflect brewing-associated pigment transformation.

Taken together, the results support an integrated interpretation of brewing-dependent quality differentiation rather than a simple “more extraction under hot brewing” model (Figure 6). Under hot-brewing conditions, elevated temperature was associated with stronger early-stage chemical responses, including higher levels of several bioactive constituent indices, broader early relative enrichment of non-volatile metabolites, and more pronounced early VOC responses. These chemical patterns coincided with darker color, stronger bitterness and astringency, and greater radical-scavenging capacity. Under cold-brewing conditions, compositional changes developed more gradually, with increasingly distinct volatile and non-volatile responses during prolonged extraction and a comparatively lighter and milder sensory profile. The resulting quality differences therefore appear to reflect heterogeneous temperature- and time-dependent compositional responses among chemical classes, potentially involving differences in mass transfer, solubilization, and compound partitioning, superimposed on the heterogeneous physicochemical properties of individual chemical classes. In this context, gossypetin 8-glucuronide, kaempferol 3-O-arabinoside, sericoside, and epoxypheophorbide a represent chemically informative markers associated with radical-scavenging-related, bitterness-related, and color dimensions, respectively, rather than proven causal drivers. Accordingly, Figure 6 should be interpreted as a proposed integrative framework for temperature- and time-dependent quality differentiation, rather than as direct evidence of specific molecular mechanisms or reaction pathways.

Several limitations should be considered when interpreting the present findings. First, HS-SPME-GC-MS annotations were based primarily on mass spectral library matching, and relative peak areas represent semi-quantitative instrumental responses; authentic-standard confirmation and targeted quantification would further strengthen compound identification and abundance estimates. Second, aroma-active contributions were not validated using odor thresholds, odor activity values, gas chromatography–olfactometry, aroma recombination, or omission experiments, and therefore the detected VOCs should not be considered confirmed key aroma compounds. Third, untargeted metabolomics primarily reflects relative abundance, and metabolite annotations not verified using authentic standards remain tentative. Fourth, the correlations between the 50 core differential metabolites and quality-related parameters were calculated using treatment means for the ten brewing conditions (*n* = 10). Although this approach is useful for exploratory integration across analytical platforms, the limited number of treatment-level observations requires cautious interpretation, and the correlations cannot establish causality. Fifth, all experiments were conducted using a single commercial batch of *C. paliurus* tea. Therefore, potential variability associated with raw-material origin, harvest batch, or processing history was not evaluated. Finally, DPPH and ABTS assays evaluate in vitro radical-scavenging responses and should not be extrapolated directly to physiological antioxidant effects. Future studies combining targeted metabolite quantification, authentic-standard confirmation, aroma-active validation, compound-specific sensory testing, and larger independent experimental datasets will be required to verify the roles of candidate metabolites and to determine whether the proposed brewing-dependent quality framework is reproducible across raw-material batches and production conditions.

## 5. Conclusions

Cold- and hot-brewing produced clearly differentiated quality profiles in *C. paliurus* tea infusions across the tested 5 min–2 h time gradient. Hot-brewed infusions exhibited a darker liquor color, stronger bitterness and astringency, higher contents of bioactive constituents, and greater in vitro DPPH and ABTS radical-scavenging capacities, whereas cold-brewed infusions presented a lighter appearance, milder taste profiles, and more gradual time-dependent compositional shifts. Volatile and non-volatile metabolomic fingerprints were clearly segregated between the two brewing modes, and their temporal patterns indicated compound-specific temperature- and time-dependent responses rather than a generalized increase in extraction efficiency at elevated temperature. Several core differential metabolite features, tentatively annotated as gossypetin 8-glucuronide, kaempferol 3-O-arabinoside, sericoside, and epoxypheophorbide a, showed significant treatment-level statistical associations with radical-scavenging capacity, bitterness-related responses, and color variation, respectively; given the exploratory correlation design and the tentative annotation level, these features should be regarded as candidate quality markers rather than confirmed causal determinants. Overall, this study characterizes how brewing temperature and time jointly differentiate the sensory, functional, and chemical quality of *C. paliurus* tea infusions and provides a chemical basis for the rational selection of brewing conditions and the development of differentiated *C. paliurus*-based herbal beverages. Because all experiments were performed with a single commercial batch of *C. paliurus* tea, these conclusions apply primarily to the tested material and brewing conditions, and their generalizability to other raw-material batches and production conditions remains to be verified.

## Figures and Tables

**Figure 1:**
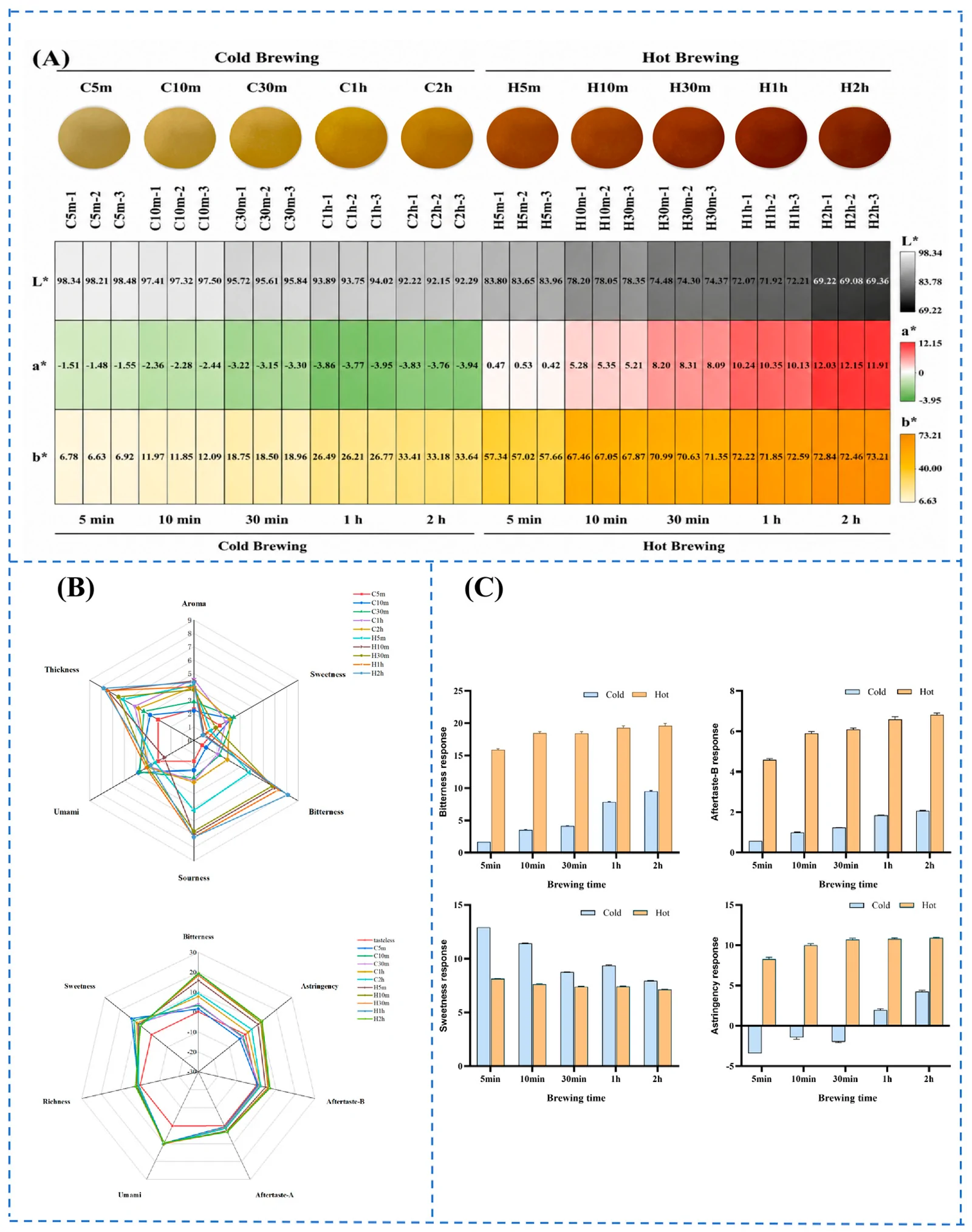
Color, sensory characteristics, and electronic tongue responses of cold- and hot-brewed *C. paliurus* tea infusions. Notes: (**A**) Visual appearance and CIELAB color parameters (L*, a*, and b*) of tea infusions under different brewing conditions. (**B**) Sensory evaluation profiles (upper) and electronic tongue response profiles (lower) of cold- and hot-brewed tea infusions. (**C**) Changes in representative electronic tongue responses, including bitterness, aftertaste-B, sweetness, and astringency, during cold and hot brewing. L*, lightness; a*, red–green coordinate; b*, yellow–blue coordinate. C and H indicate cold and hot brewing, respectively; 5 m, 10 m, 30 m, 1 h, and 2 h indicate brewing times of 5 min, 10 min, 30 min, 1 h, and 2 h, respectively.

**Figure 2:**
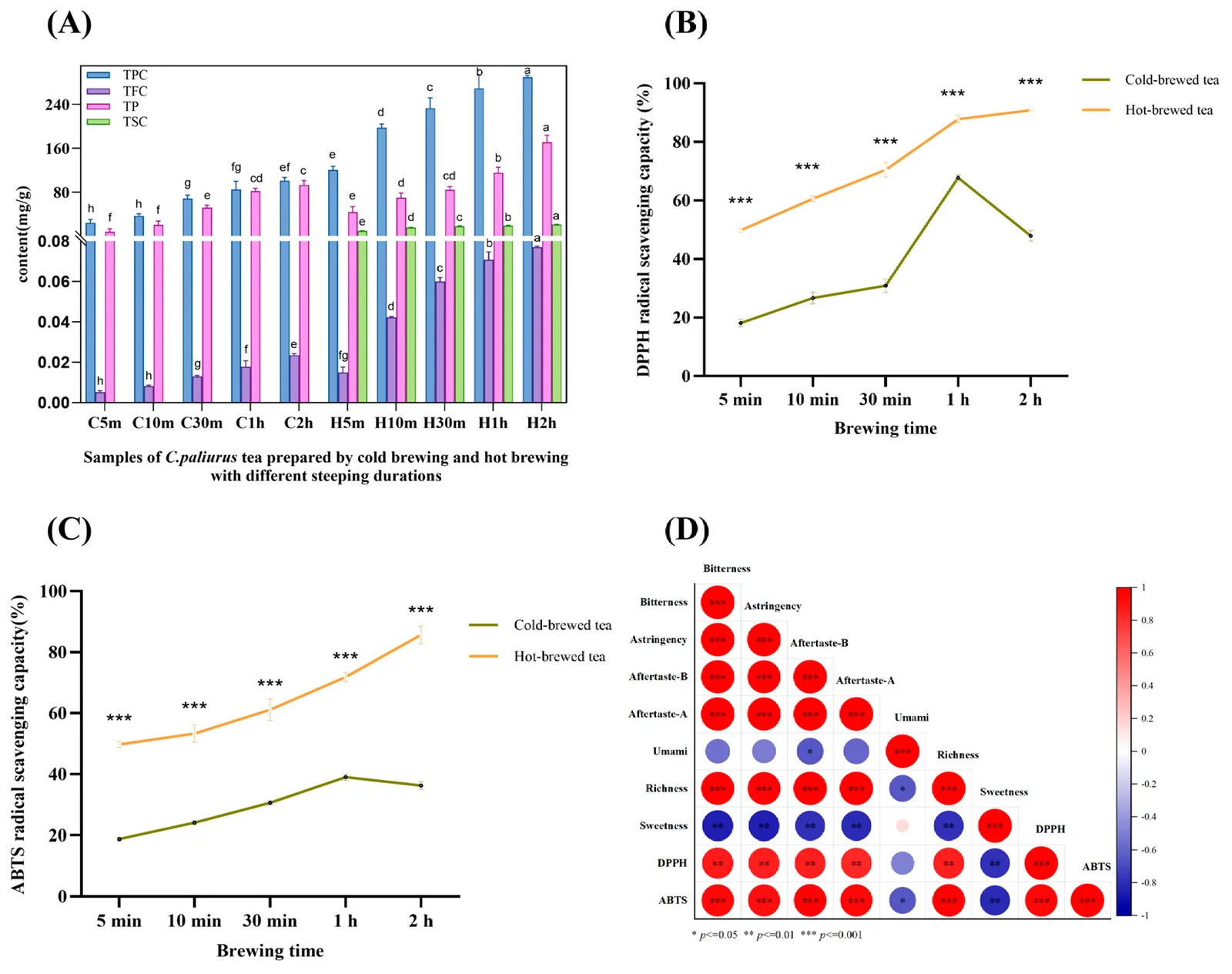
Bioactive constituents, radical-scavenging capacities, and their associations with electronic tongue responses in cold- and hot-brewed *C. paliurus* tea infusions. Notes: (**A**) Total phenolic content (TPC), total flavonoid content (TFC), total polysaccharide content (TP), and total saponin content (TSC). (**B**) DPPH radical-scavenging capacity. (**C**) ABTS radical-scavenging capacity. (**D**) Pearson correlation matrix of electronic tongue responses and radical-scavenging capacities. Data in (**A**–**C**) are presented as mean ± SD (*n* = 3 independent brewing replicates). In (**A**), different lowercase letters indicate significant differences among brewing treatments for the same bioactive constituent according to Duncan’s multiple-range test (*p* < 0.05). In (**B**,**C**), asterisks indicate significant differences between cold- and hot-brewed samples at the same brewing time (*** *p* < 0.001). In (**D**), red and blue circles indicate positive and negative correlations, respectively; circle size represents |r|, and asterisks indicate significance levels (* *p* < 0.05, ** *p* < 0.01, *** *p* < 0.001).

**Figure 3:**
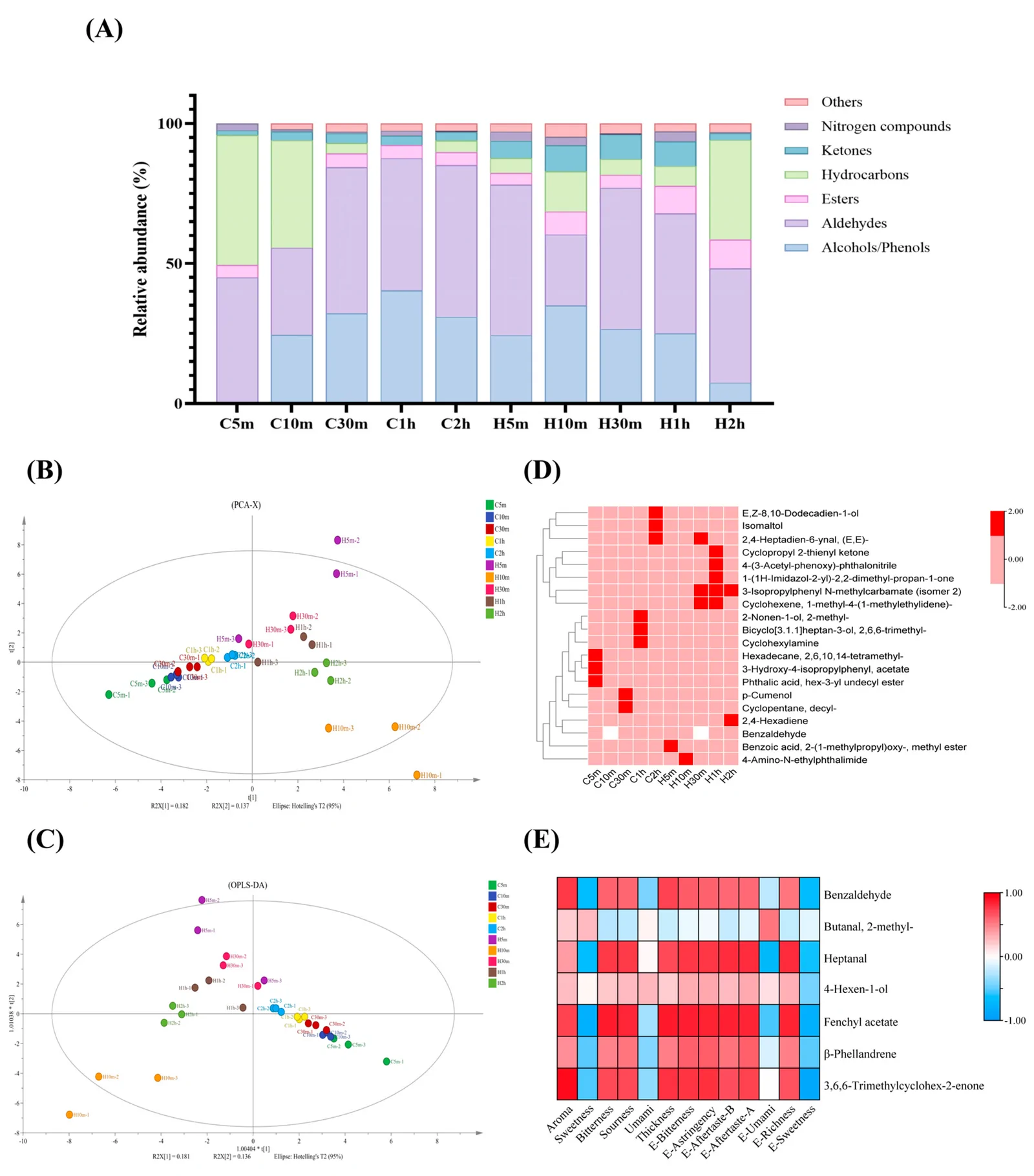
Volatile profiles and their associations with sensory characteristics of cold- and hot-brewed *C. paliurus* tea infusions. Notes: (**A**) Relative abundance of different volatile compound classes. (**B**) PCA score plot of GC-MS volatile profiles. (**C**) OPLS-DA score plot of GC-MS volatile profiles. (**D**) Hierarchical clustering heatmap of the top 20 VOCs ranked by VIP values. (**E**) Pearson correlation heatmap between seven representative VOCs and sensory attributes and electronic tongue responses. The representative VOCs were selected based on differences between cold- and hot-brewed samples, detection frequency, temporal response patterns, and chemical-class representativeness. In (**D**), data were log10-transformed and row-wise Z-score normalized; red and blue indicate relatively high and low levels, respectively. In (**E**), red and blue indicate positive and negative correlations, respectively. C and H indicate cold and hot brewing, respectively; 5 m, 10 m, 30 m, 1 h, and 2 h indicate brewing times of 5 min, 10 min, 30 min, 1 h, and 2 h, respectively.

**Figure 4:**
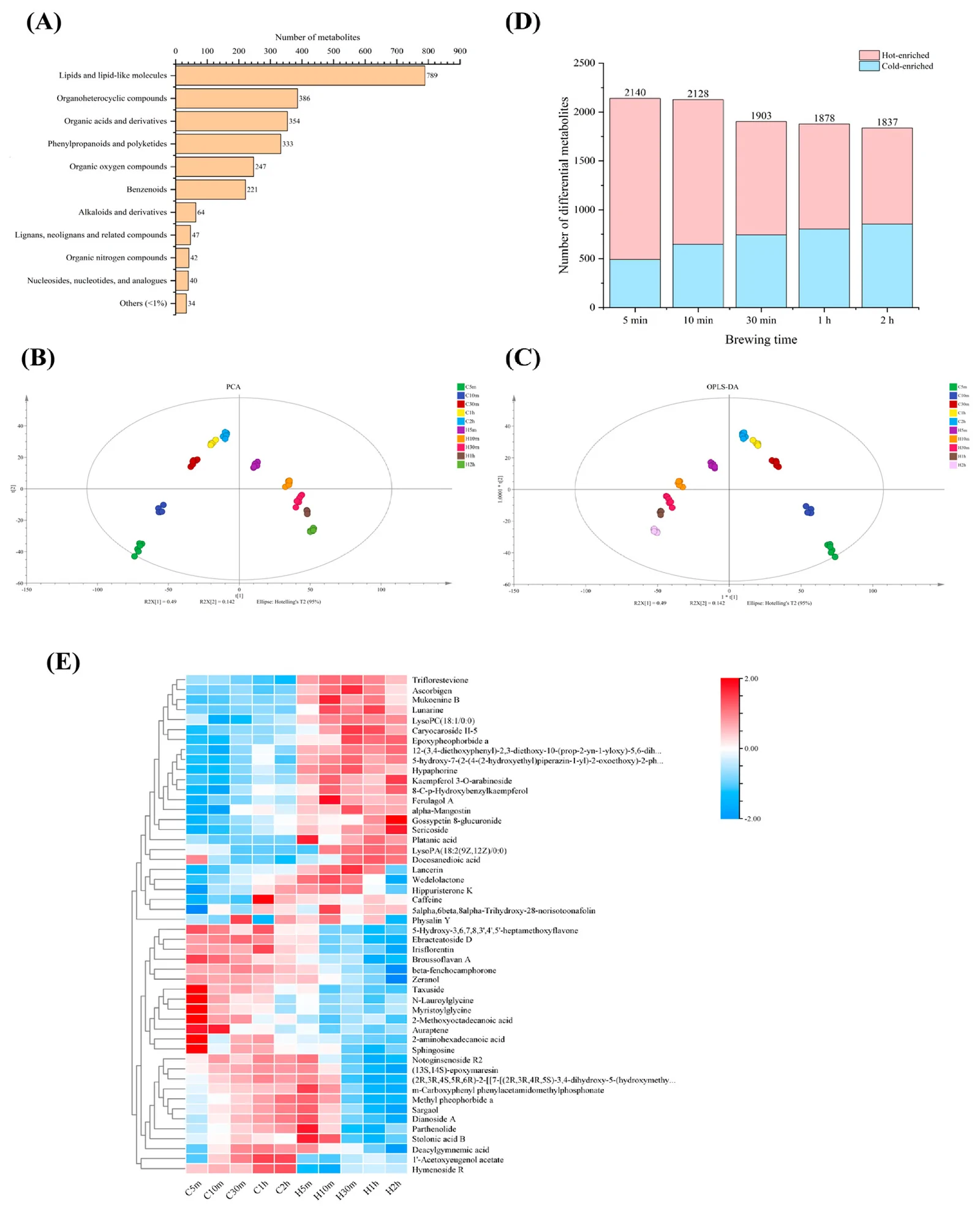
Untargeted metabolomic profiles and differential metabolite responses of cold- and hot-brewed *C. paliurus* tea infusions. Notes: (**A**) Superclass distribution of classified metabolite features. (**B**) PCA score plot based on the overall metabolite profiles. (**C**) OPLS-DA score plot of cold- and hot-brewed samples. (**D**) Numbers of differential metabolite features showing relatively higher abundance under cold- or hot-brewing conditions at matched brewing times. (**E**) Hierarchical clustering heatmap of 50 core differential metabolite features. PCA and OPLS-DA were performed using 3666 database-annotated metabolite features. In (**A**), 2557 classified features were included, whereas 1109 unclassified features were excluded. The 50 core differential metabolite features in (**E**) were selected based on VIP > 1, FDR < 0.05, |log_2_FC| > 1, and consistent response patterns across matched-time comparisons. C and H indicate cold and hot brewing, respectively; 5 m, 10 m, 30 m, 1 h, and 2 h indicate brewing times of 5 min, 10 min, 30 min, 1 h, and 2 h, respectively.

**Figure 5:**
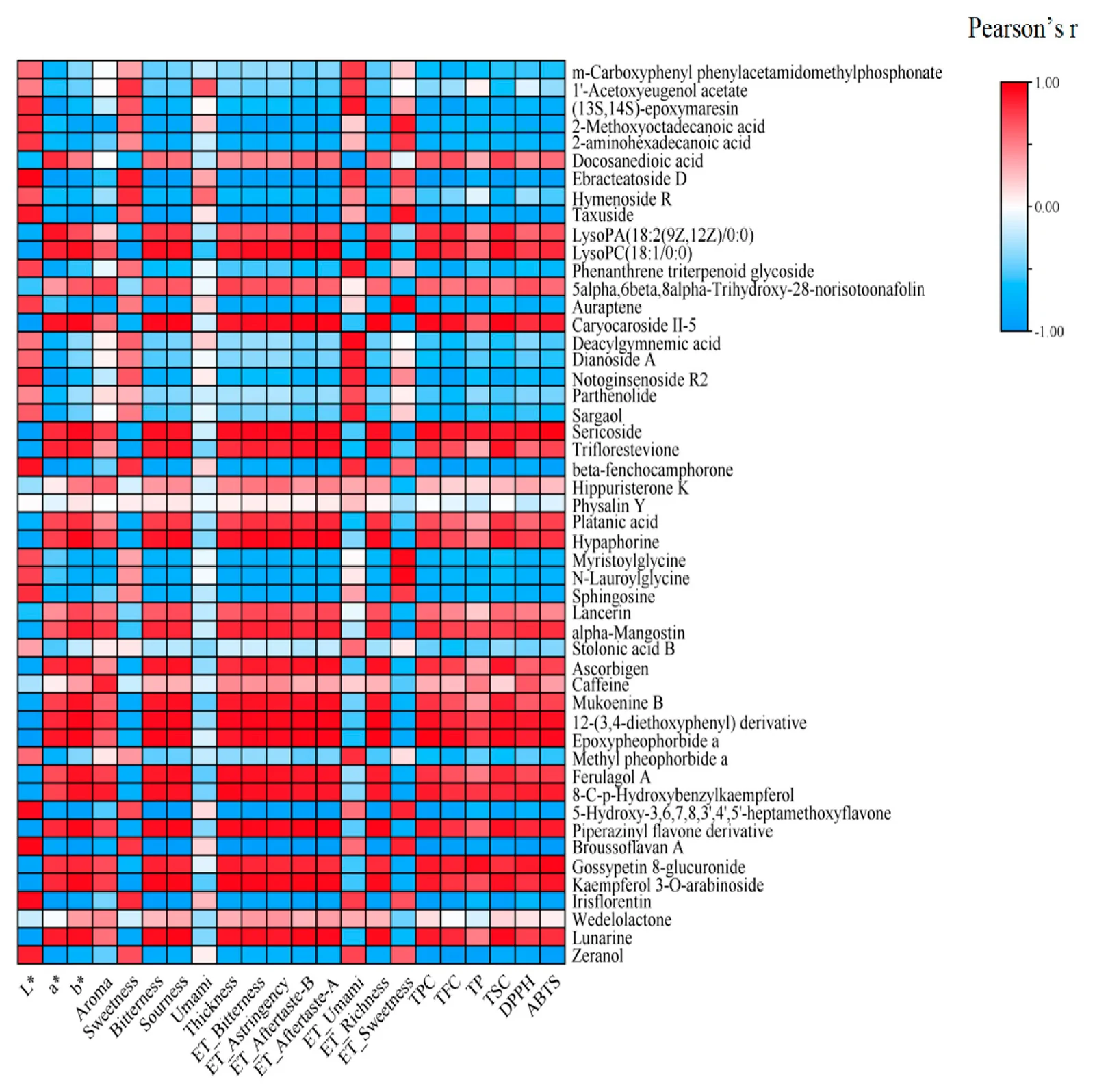
Correlations between core differential metabolites and quality characteristics of cold- and hot-brewed *C. paliurus* tea infusions. Notes: Pearson correlations were calculated between 50 core differential metabolites and 22 quality-related parameters, including color parameters (L*, a*, and b*), sensory attributes, electronic tongue responses, bioactive constituents (TPC, TFC, TP, and TSC), and radical-scavenging capacities (DPPH and ABTS), using the mean values of the 10 brewing treatments (*n* = 10). Red and blue indicate positive and negative correlations, respectively, and color intensity represents Pearson’s r (−1 to 1). E- denotes electronic tongue responses. *p* values were adjusted for multiple comparisons using the Benjamini–Hochberg FDR procedure, and q values were used to assess statistical significance.

**Figure 6:**
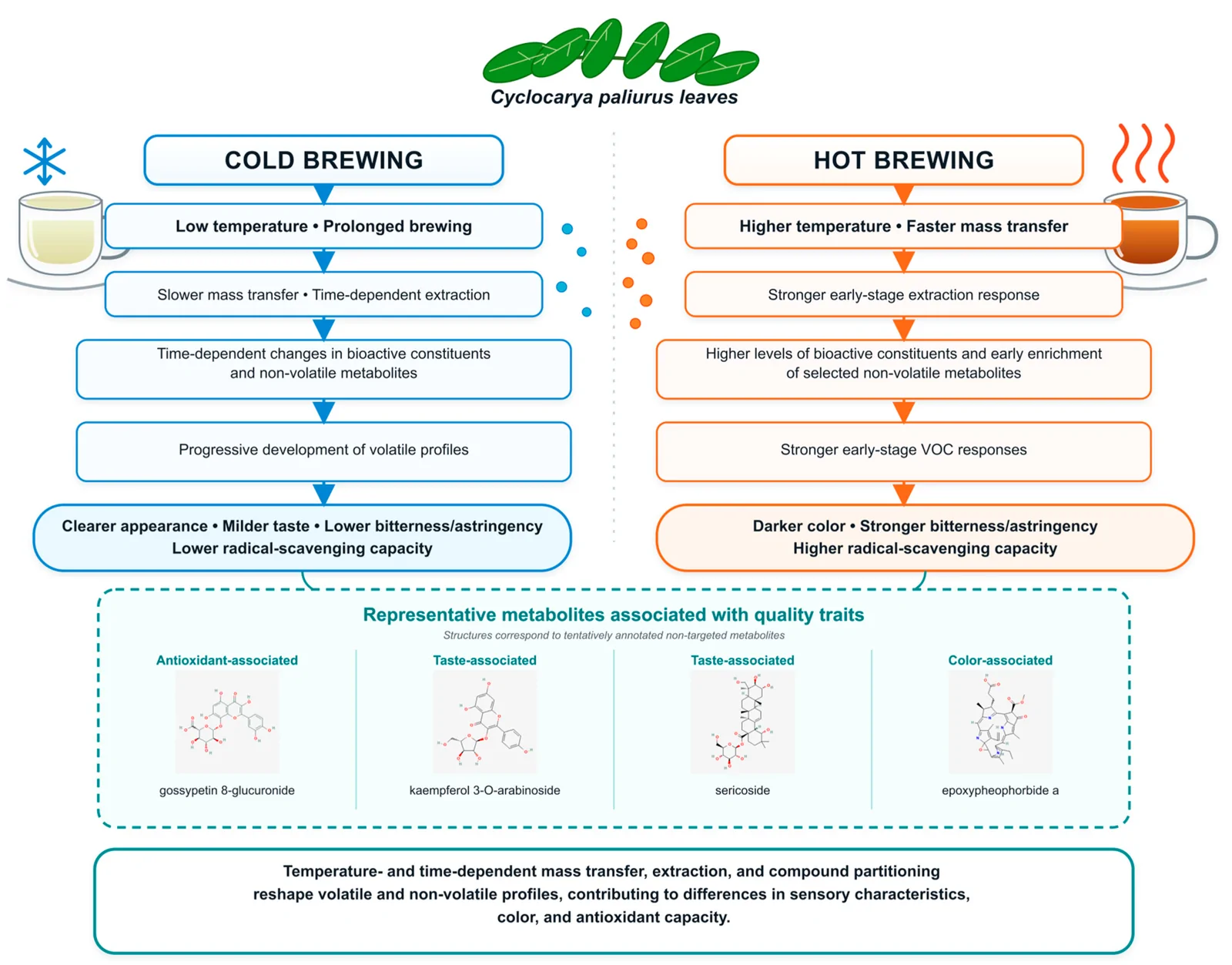
Proposed integrative framework for temperature- and time-dependent quality differentiation of cold- and hot-brewed *C. paliurus* tea infusions. Notes: The schematic summarizes brewing-associated differences in apparent extraction responses, bioactive constituent levels, volatile and non-volatile chemical profiles, sensory characteristics, infusion color, and radical-scavenging capacity. Representative metabolites associated with antioxidant-related, taste-related, and color-related traits are highlighted, and the depicted structures correspond to tentatively annotated metabolites from the untargeted metabolomics dataset.

## Data Availability

The original contributions presented in the study are included in the article/Supplementary Materials; further inquiries can be directed to the corresponding author.

## Supplementary Materials

The following supporting information can be downloaded at: https://www.mdpi.com/article/10.3390/foods15203691/s1, Figure S1: Total ion chromatograms (TICs) obtained by HS-SPME-GC-MS analysis of cold- and hot-brewed *C. paliurus* tea infusions; Figure S2: Quality-control assessment of the untargeted metabolomics dataset; Figure S3: Temperature- and time-dependent variation in the number of detected volatile features in cold- and hot-brewed *C. paliurus* tea infusions. Table S1. Panelist-to-panelist variability in the sensory evaluation of cold- and hot-brewed *C. paliurus* tea infusions; Table S2: Electronic tongue response values of cold- and hot-brewed *C. paliurus* tea infusions under different brewing conditions; Table S3: Contents of bioactive components in cold- and hot-brewed *C. paliurus* tea infusions; Table S4: DPPH and ABTS radical-scavenging capacities of cold- and hot-brewed *C. paliurus* tea infusions; Table S5: Volatile compounds tentatively annotated in cold- and hot-brewed *C. paliurus* tea infusions by HS-SPME-GC-MS; Table S6: Classification, tentative annotation, and statistical information of the 50 core differential metabolite features selected from cold- and hot-brewed *C. paliurus* tea infusions; Table S7: Two-way ANOVA and HC3 heteroscedasticity-robust interaction analysis of 50 representative differential metabolite features in cold- and hot-brewed *C. paliurus* tea infusions.

## References

[1] Li Y., Quan H., Liang L., Yang T., Feng L., Mao X., Wang Y. (2021). Nontargeted metabolomics reveals the discrimination of *Cyclocarya paliurus* leaves brewed by different methods. Food Res. Int..

[2] Liang L., Liu Y., Liu Y., Gan S., Mao X., Wang Y. (2022). Untargeted metabolomics analysis based on HS-SPME-GC-MS and UPLC-Q-TOF/MS reveals the contribution of stem to the flavor of *Cyclocarya paliurus* herbal extract. LWT.

[3] Chen L., Liu F., Yang Y., Tu Z., Lin J., Ye Y., Xu P. (2021). Oxygen-enriched fermentation improves the taste of black tea by reducing the bitter and astringent metabolites. Food Res. Int..

[4] Zhou B., Ma B., Xu C., Wang J., Wang Z., Huang Y., Ma C. (2022). Impact of enzymatic fermentation on taste, chemical compositions and in vitro antioxidant activities in Chinese teas using E-tongue, HPLC and amino acid analyzer. LWT.

[5] Zhang H., Zhang J., Liu S., Li T., Wei Y., Gu Z., Su Z., Ning J., Wang Y., Hou Z. (2024). Characterization of the key volatile compounds in Longjing tea (*Camellia sinensis*) with different aroma types at different steeping temperatures by GC-MS and GC-IMS. LWT.

[6] Wang H., Hua J., Yu Q., Li J., Wang J., Deng Y., Yuan H., Jiang Y. (2021). Widely targeted metabolomic analysis reveals dynamic changes in non-volatile and volatile metabolites during green tea processing. Food Chem..

[7] Yu J., Liu Y., Zhang S., Luo L., Zeng L. (2021). Effect of brewing conditions on phytochemicals and sensory profiles of black tea infusions: A primary study on the effects of geraniol and β-ionone on taste perception of black tea infusions. Food Chem..

[8] Li M., Feng Z., Wang F., Chen J., Fan J., Wang J., Liu Z., Yin J. (2023). Effects of brewing water on the volatile composition of tea infusions. Food Chem..

[9] Xiao Y., He C., Chen Y., Ho C.-T., Wu X., Huang Y., Gao Y., Hou A., Li Z., Wang Y. (2022). UPLC–QQQ–MS/MS-based widely targeted metabolomic analysis reveals the effect of solid-state fermentation with *Eurotium cristatum* on the dynamic changes in the metabolite profile of dark tea. Food Chem..

[10] Yang J., Guo J., Yuan J. (2008). In vitro antioxidant properties of rutin. LWT-Food Sci. Technol..

[11] Wu L., Gao Y., Ren W., Su Y., Li J., Du Y., Wang Q., Kuang H. (2022). Rapid determination and origin identification of total polysaccharides contents in *Schisandra chinensis* by near-infrared spectroscopy. Spectrochim. Acta A Mol. Biomol. Spectrosc..

[12] de Aguiar N.S., Hansel F.A., Reis C.A.F., Lazzarotto M., Wendling I. (2024). Optimizing the vanillin-acid sulfuric method to total saponin content in leaves of yerba mate clones. Chem. Biodivers..

[13] Brand-Williams W., Cuvelier M.E., Berset C. (1995). Use of a free radical method to evaluate antioxidant activity. LWT-Food Sci. Technol..

[14] Re R., Pellegrini N., Proteggente A., Pannala A., Yang M., Rice-Evans C. (1999). Antioxidant activity applying an improved ABTS radical cation decolorization assay. Free Radic. Biol. Med..

[15] Lin S.-D., Yang J.-H., Hsieh Y.-J., Liu E.-H., Mau J.-L. (2014). Effect of different brewing methods on quality of green tea. J. Food Process. Preserv..

[16] Yang C., Wang Z., Xu M., Wei K., Dai Q., Wan X., Leong O., Lin R., Cui C., Hou R. (2025). The chemical basis of aroma/taste and color formation in green tea infusion during cold brewing revealed by metabolomics analysis. Food Chem..

[17] He W., Hu X., Zhao L., Liao X., Zhang Y., Zhang M., Wu J. (2009). Evaluation of Chinese tea by the electronic tongue: Correlation with sensory properties and classification according to geographical origin and grade level. Food Res. Int..

[18] Chen L., Lu D., Wan Y., Zou Y., Zhang R., Zhou T., Long B., Zhu K., Wang W., Tian X. (2024). Metabolite profiling and identification of sweet/bitter taste compounds in the growth of *Cyclocarya paliurus* leaves using multiplatform metabolomics. Foods.

[19] Liu Y., Luo L., Liao C., Chen L., Wang J., Zeng L. (2018). Effects of brewing conditions on the phytochemical composition, sensory qualities and antioxidant activity of green tea infusion: A study using response surface methodology. Food Chem..

[20] Xie J.-H., Dong C.-J., Nie S.-P., Li F., Wang Z.-J., Shen M.-Y., Xie M.-Y. (2015). Extraction, chemical composition and antioxidant activity of flavonoids from *Cyclocarya paliurus* (Batal.) Iljinskaja leaves. Food Chem..

[21] Zhou M., Lin Y., Fang S., Liu Y., Shang X. (2019). Phytochemical content and antioxidant activity in aqueous extracts of *Cyclocarya paliurus* leaves collected from different populations. PeerJ.

[22] Lou J., Zou Y., Tian X. (2024). Characterization and analysis of the volatile components of *Cyclocarya paliurus* tea from different origins based on GC-IMS technology. Food Mach..

[23] Bai M., Wang L. (2024). Untargeted metabolomics analysis reveals the effect of fixation on the profile of volatile compounds of *Cyclocarya paliurus* tea. Pol. J. Food Nutr. Sci..

[24] Sun L., Dong X., Ren Y., Agarwal M., Ren A., Ding Z. (2022). Profiling real-time aroma from green tea infusion during brewing. Foods.

[25] Zhu Y., Lv H.-P., Shao C.-Y., Kang S., Zhang Y., Guo L., Dai W.-D., Tan J.-F., Peng Q.-H., Lin Z. (2018). Identification of key odorants responsible for chestnut-like aroma quality of green teas. Food Res. Int..

[26] Yin P., Kong Y.-S., Liu P.-P., Jiang C.-L., Sun M.-F., Guo G.-Y., Liu Z.-H. (2022). Temporal variation of the non-volatile compounds and key odorants in Xinyang Maojian green teas during the spring and autumn seasons. Agronomy.

[27] Zheng X., Xiao H., Chen J., Zhu J., Fu Y., Ouyang S., Chen Y., Chen D., Su J., Xue T. (2022). Metabolome and whole-transcriptome analyses reveal the molecular mechanisms underlying hypoglycemic nutrient metabolites biosynthesis in *Cyclocarya paliurus* leaves during different harvest stages. Front. Nutr..

[28] Fu Y., Zou Y., Tian X. (2025). Analysis of flavor characteristics and bioactive components of *Cyclocarya paliurus* tea from different origins based on electronic tongue and HS-SPME-GC-MS technology. Sci. Technol. Food Ind..

[29] Sumner L.W., Amberg A., Barrett D., Beale M.H., Beger R., Daykin C.A., Fan T.W.-M., Fiehn O., Goodacre R., Griffin J.L. (2007). Proposed minimum reporting standards for chemical analysis. Metabolomics.

[30] Schymanski E.L., Jeon J., Gulde R., Fenner K., Ruff M., Singer H.P., Hollender J. (2014). Identifying small molecules via high resolution mass spectrometry: Communicating confidence. Environ. Sci. Technol..

[31] Spiegel M., Kowalczyk A. (2025). Aglycone, glycoside, or glucuronide? Experimental and mechanistic insights into the antioxidative potential of gossypetin, gossypin, and hibifolin. J. Phys. Chem. B.

[32] Walser C., Spaccasassi A., Gradl K., Stark T.D., Sterneder S., Wolter F.P., Achatz F., Frank O., Somoza V., Hofmann T. (2024). Human sensory, taste receptor, and quantitation studies on kaempferol glycosides derived from rapeseed/canola protein isolates. J. Agric. Food Chem..

[33] Zhu L., Pan Y., Li Y., Zhou Y., Liu H., Liu X. (2024). Molecular mechanisms of bitterness and astringency in the oral cavity induced by soyasaponin. Food Sci. Hum. Wellness.

[34] Yin J.-F., Zhang Y.-N., Du Q.-Z., Chen J.-X., Yuan H.-B., Xu Y.-Q. (2020). Association between chemistry and taste of tea: A review. Trends Food Sci. Technol..

[35] Lu J.-L., Pan S.-S., Zheng X.-Q., Dong J.-J., Borthakur D., Liang Y.-R. (2009). Effects of lipophilic pigments on colour of the green tea infusion. Int. J. Food Sci. Technol..

[36] Saijō R. (1970). Degradation of chlorophylls in tea leaves by fermentation and heating. Tea Res. J..

