# Supplementary material for: Effects of Brewing Temperature and Time on the Quality Attributes and Chemical Profiles of Cyclocarya paliurus Tea Infusions
Source: Foods. 2026 Oct 16;15(20):3691. doi: 10.3390/foods15203691 (PMC13703686; doi:10.3390/foods15203691)
Supplement: Supplementary file 1 [file foods-15-03691-s001.zip › foods-4631265-supplementary.pdf]

Supplementary Material

Figure

Figure S1. Total ion chromatograms (TICs) obtained by HS-SPME-GC-MS analysis of cold- and hot-brewed *C. paliurus* tea infusions

(A) C5m

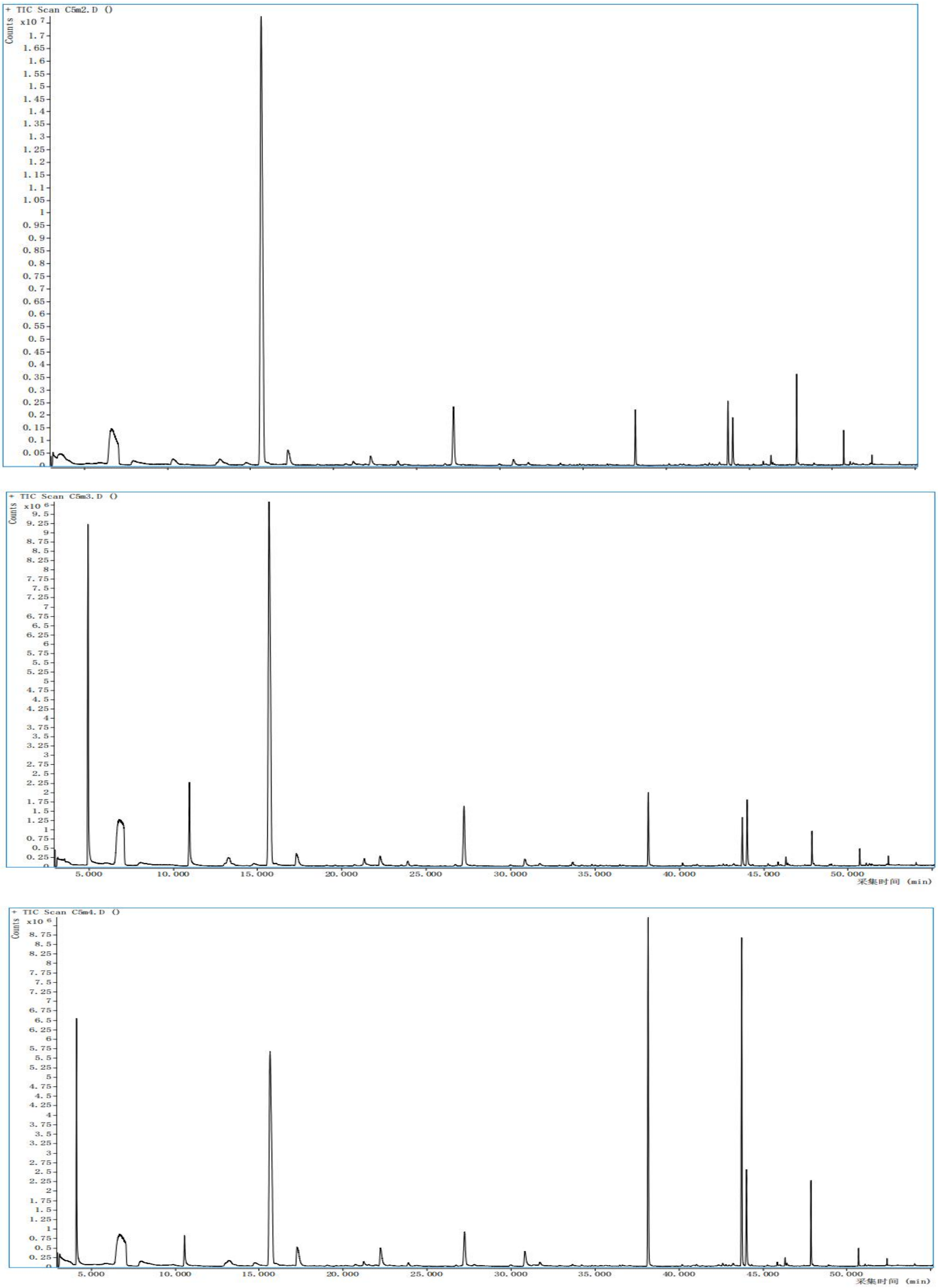

(B) C10m

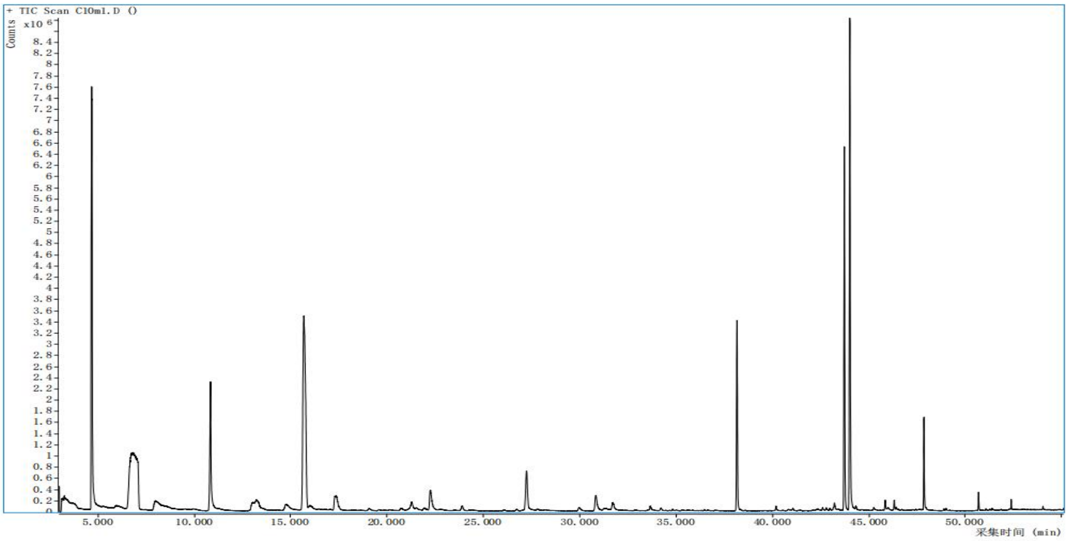
+ TIC Scan C10m1.D ()
Counts
x10 6
采集时间 (min)

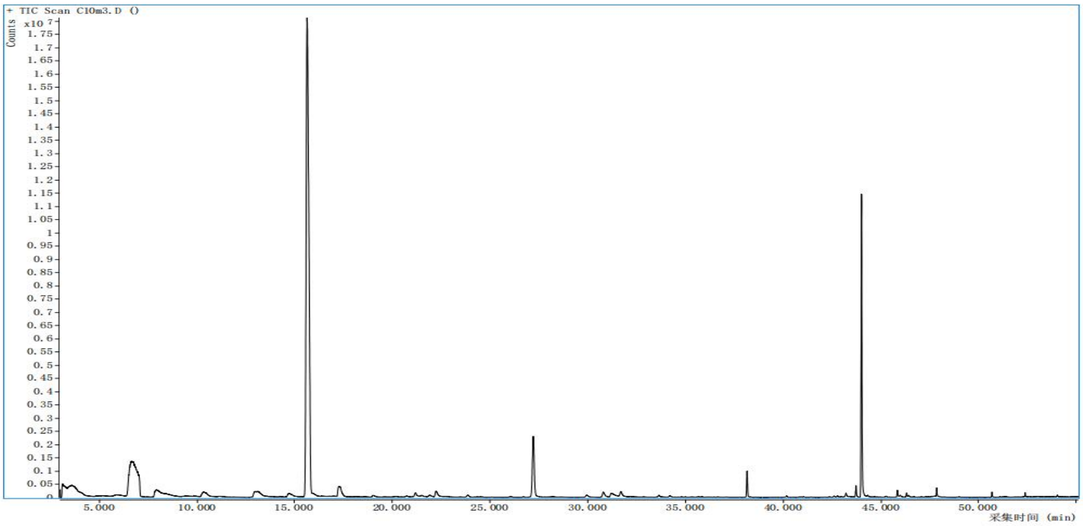
+ TIC Scan C10m3.D ()
Counts
x10 7
采集时间 (min)

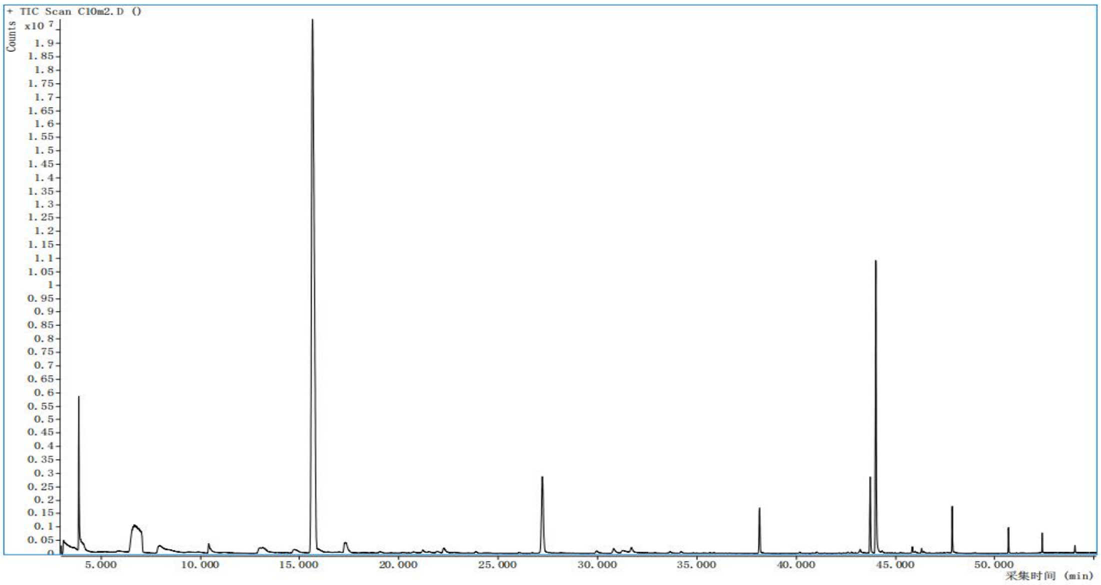
+ TIC Scan C10m2.D ()
Counts
x10 7
采集时间 (min)

(C) C30m

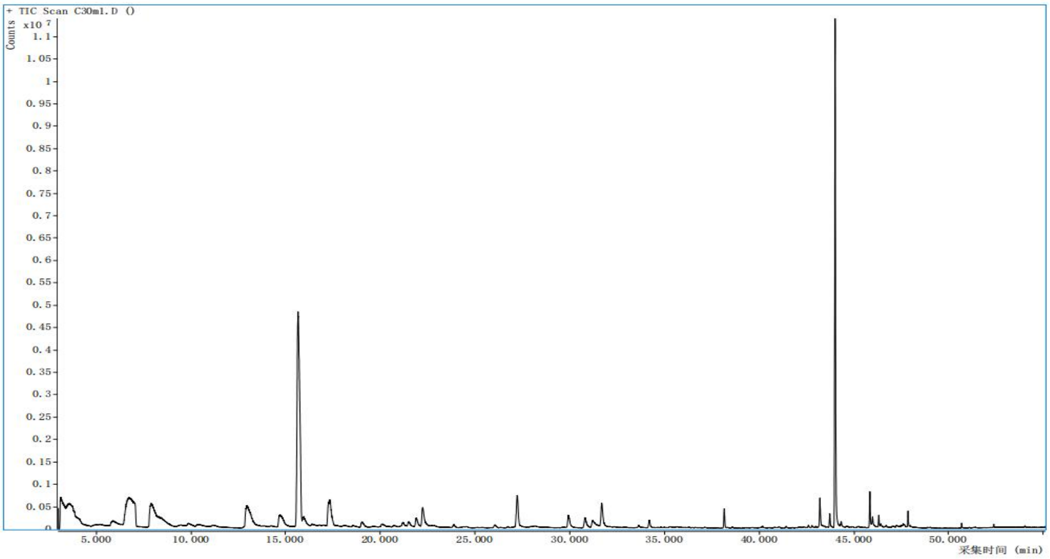
+ TIC Scan C30m1.D ()
Counts x10 7
采集时间 (min)

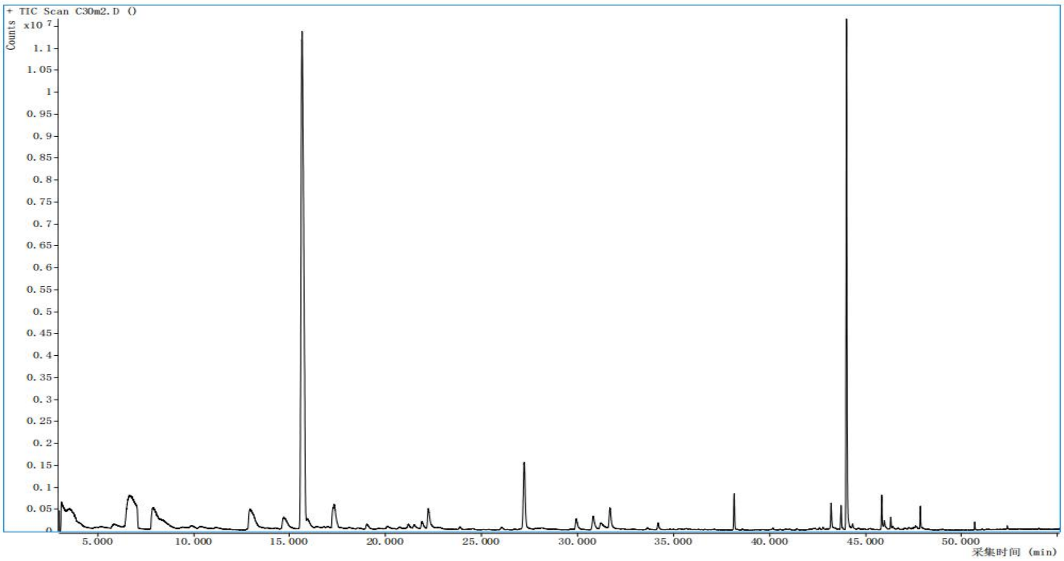
+ TIC Scan C30m2.D ()
Counts x10 7
采集时间 (min)

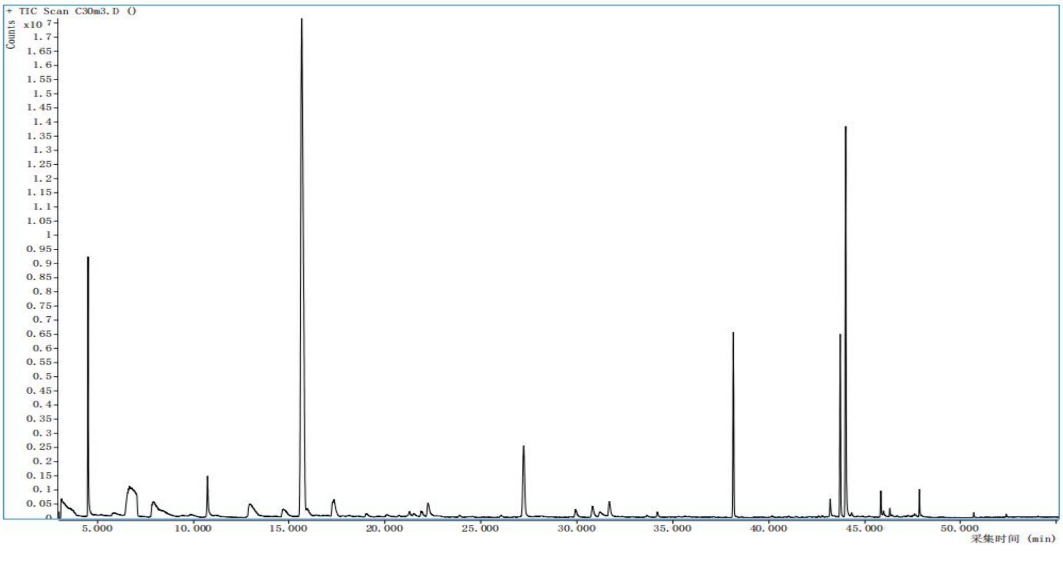
+ TIC Scan C30m3.D ()
Counts x10 7
采集时间 (min)

(D) C1h

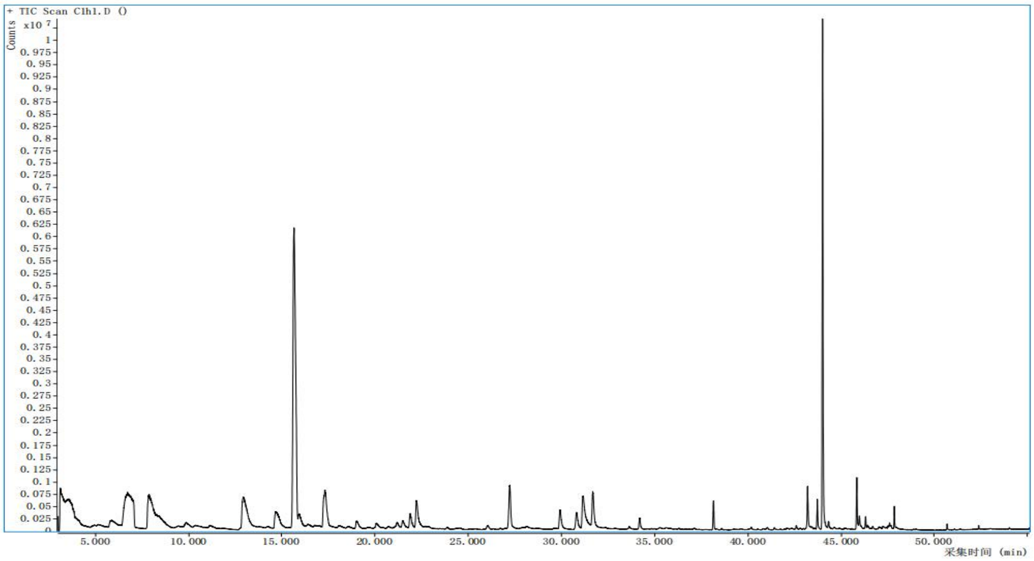
+ TIC Scan C1h1.D ()
Counts x10 7
采集时间 (min)

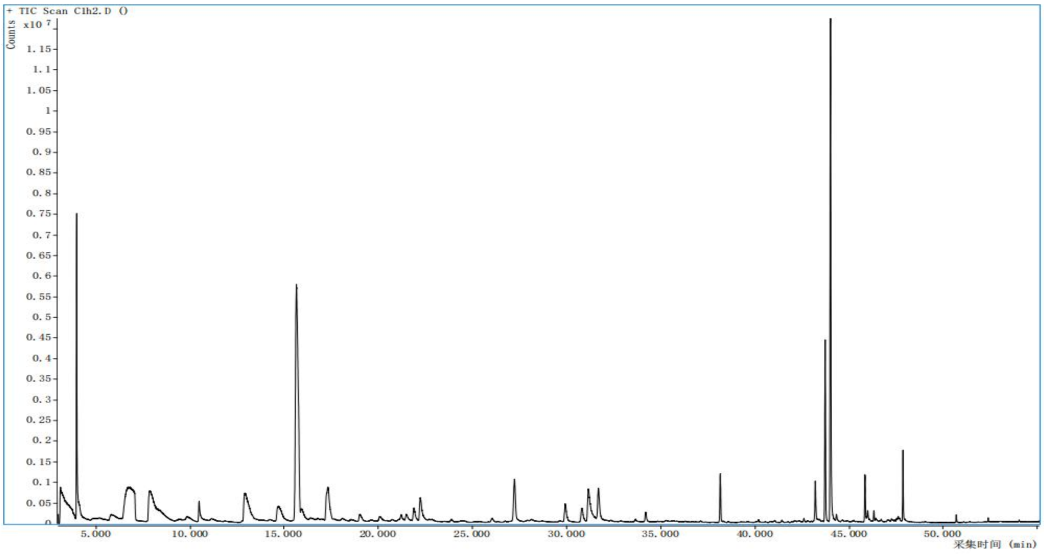
+ TIC Scan C1h2.D ()
Counts x10 7
采集时间 (min)

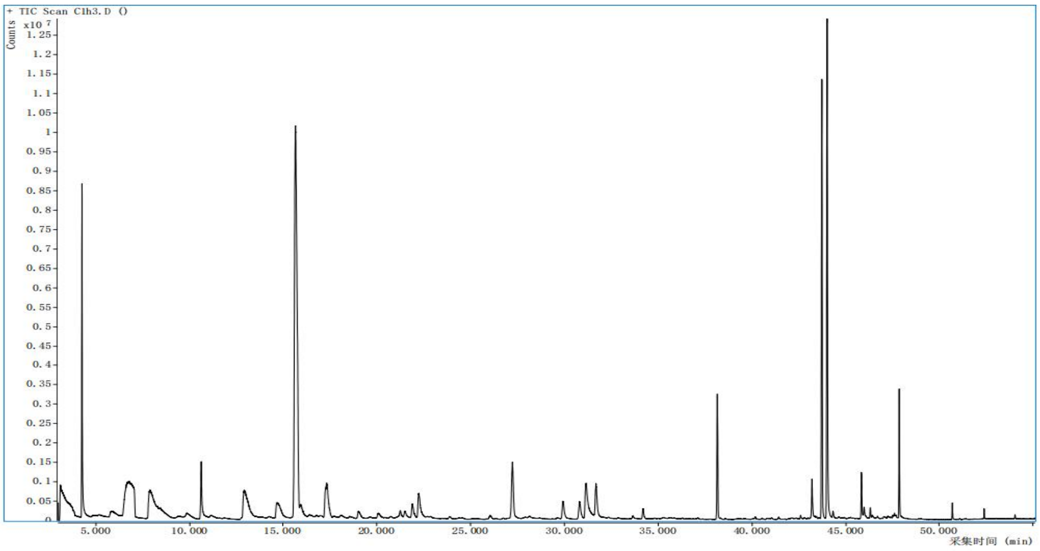
+ TIC Scan C1h3.D ()
Counts x10 7
采集时间 (min)

(E) C2h

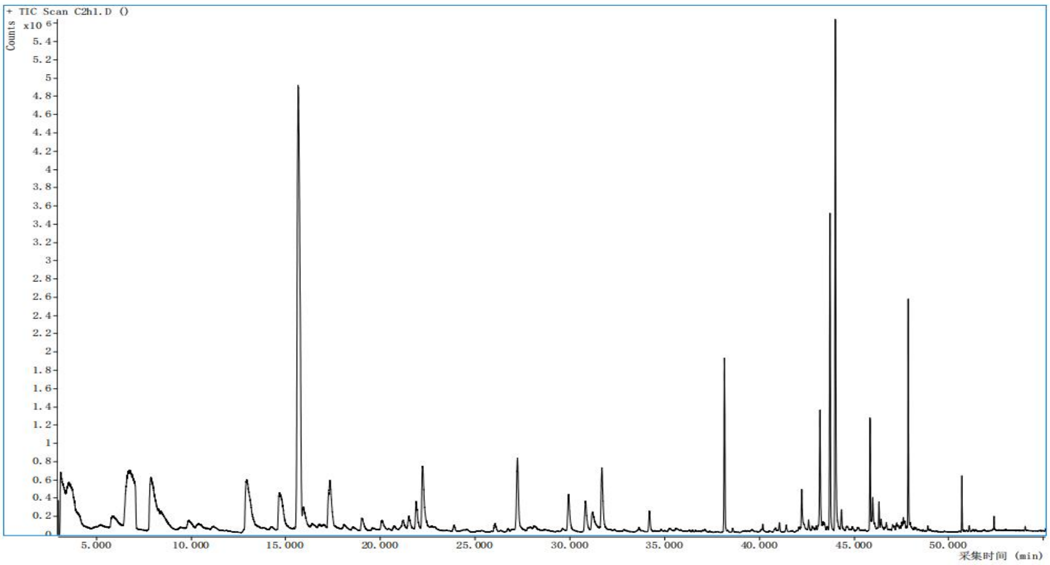
+ TIC Scan C2h1.D ()
Counts
x10 6
采集时间 (min)

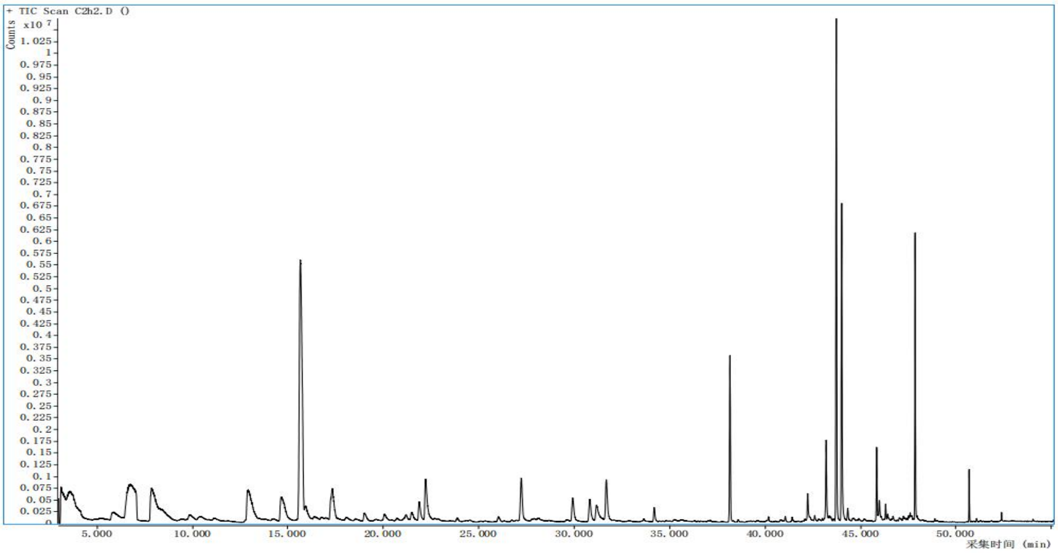
+ TIC Scan C2h2.D ()
Counts
x10 7
采集时间 (min)

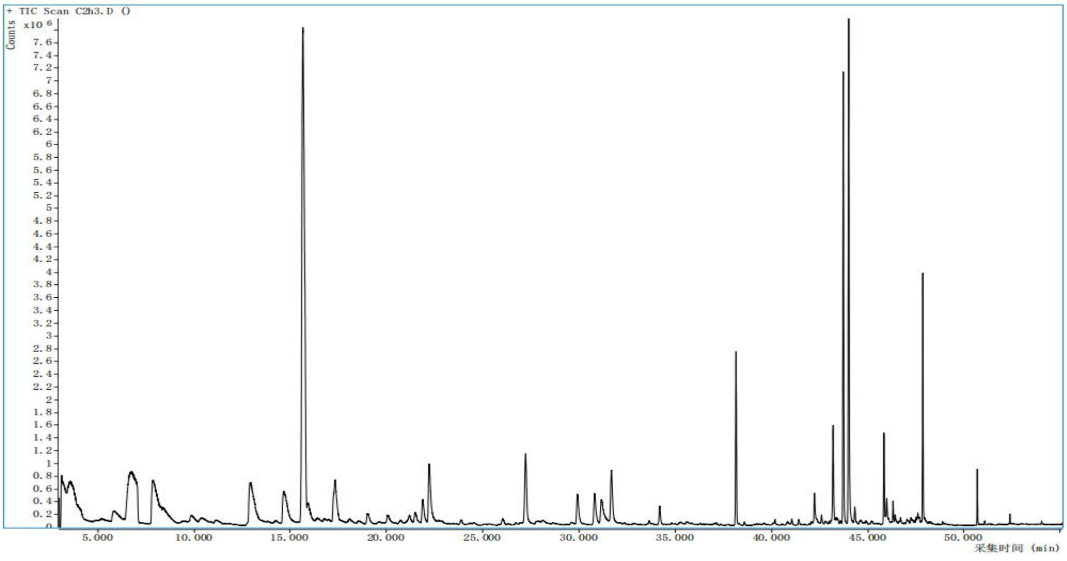
+ TIC Scan C2h3.D ()
Counts
x10 6
采集时间 (min)

(F) H5m

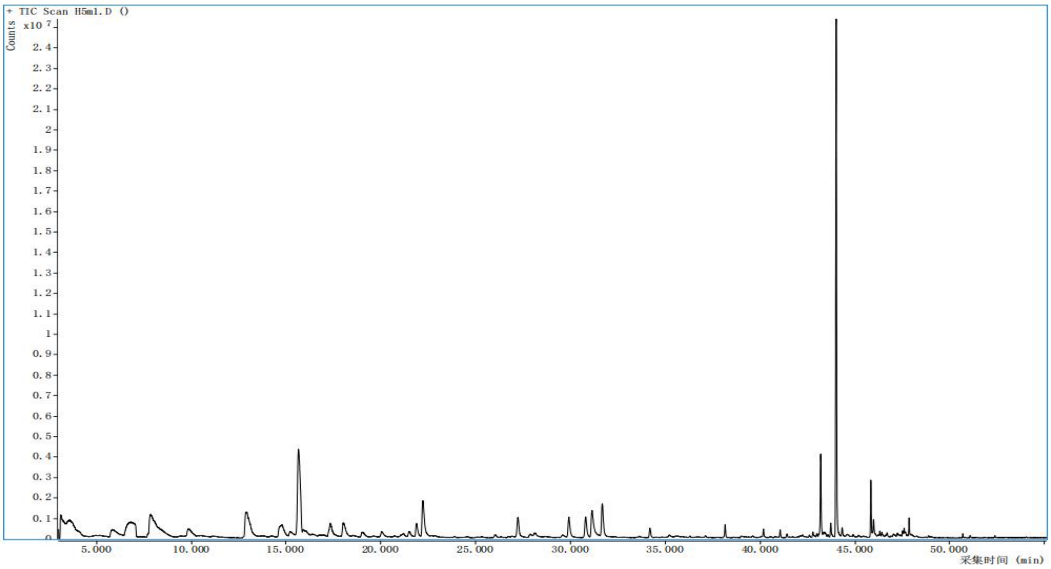

+ TIC Scan H5m1.D ()
Counts
x10 7
2.4
2.3
2.2
2.1
2
1.9
1.8
1.7
1.6
1.5
1.4
1.3
1.2
1.1
1
0.9
0.8
0.7
0.6
0.5
0.4
0.3
0.2
0.1
0
5.000
10.000
15.000
20.000
25.000
30.000
35.000
40.000
45.000
50.000
采集时间 (min)

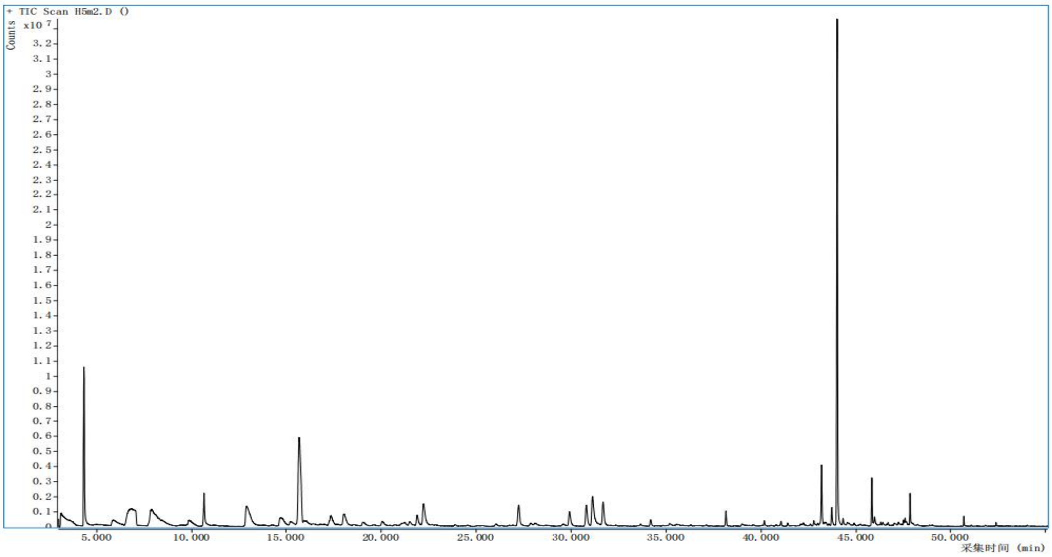

+ TIC Scan H5m2.D ()
Counts
x10 7
3.2
3.1
3
2.9
2.8
2.7
2.6
2.5
2.4
2.3
2.2
2.1
2
1.9
1.8
1.7
1.6
1.5
1.4
1.3
1.2
1.1
1
0.9
0.8
0.7
0.6
0.5
0.4
0.3
0.2
0.1
0
5.000
10.000
15.000
20.000
25.000
30.000
35.000
40.000
45.000
50.000
采集时间 (min)

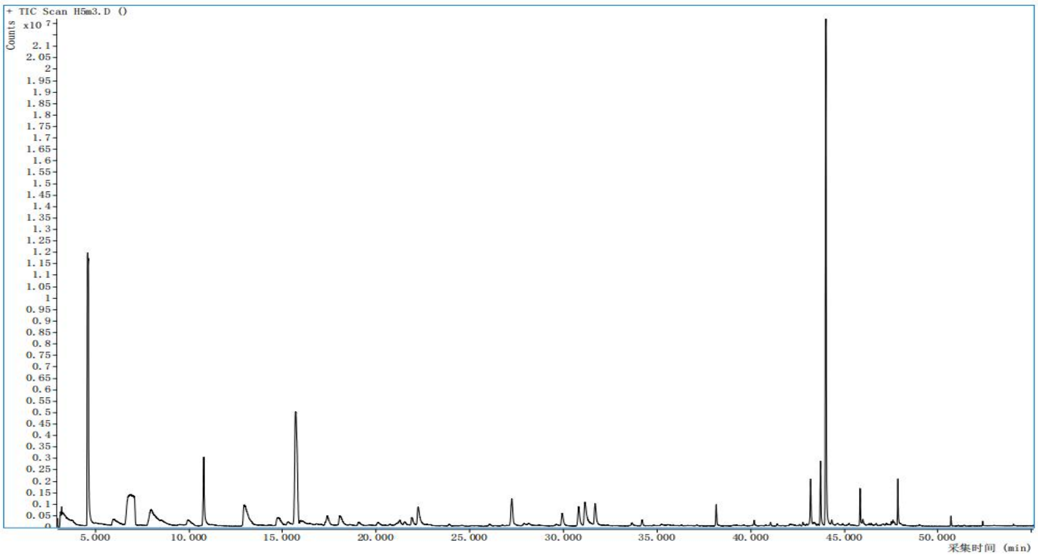

+ TIC Scan H5m3.D ()
Counts
x10 7
2.1
2.05
2
1.95
1.9
1.85
1.8
1.75
1.7
1.65
1.6
1.55
1.5
1.45
1.4
1.35
1.3
1.25
1.2
1.15
1.1
1.05
1
0.95
0.9
0.85
0.8
0.75
0.7
0.65
0.6
0.55
0.5
0.45
0.4
0.35
0.3
0.25
0.2
0.15
0.1
0.05
0
5.000
10.000
15.000
20.000
25.000
30.000
35.000
40.000
45.000
50.000
采集时间 (min)

(G) H10m

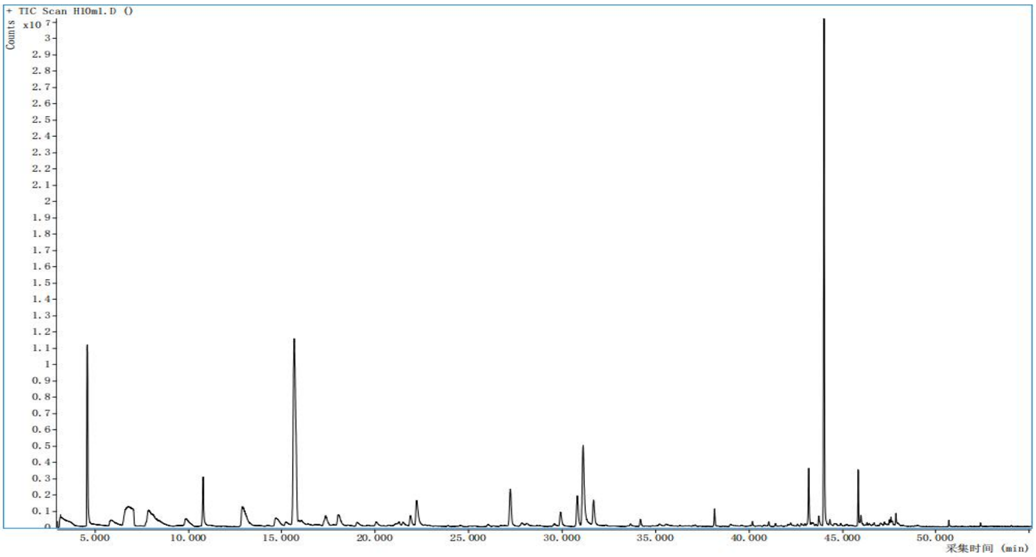
+ TIC Scan H10m1.D ()
Counts
x10 7
采集时间 (min)

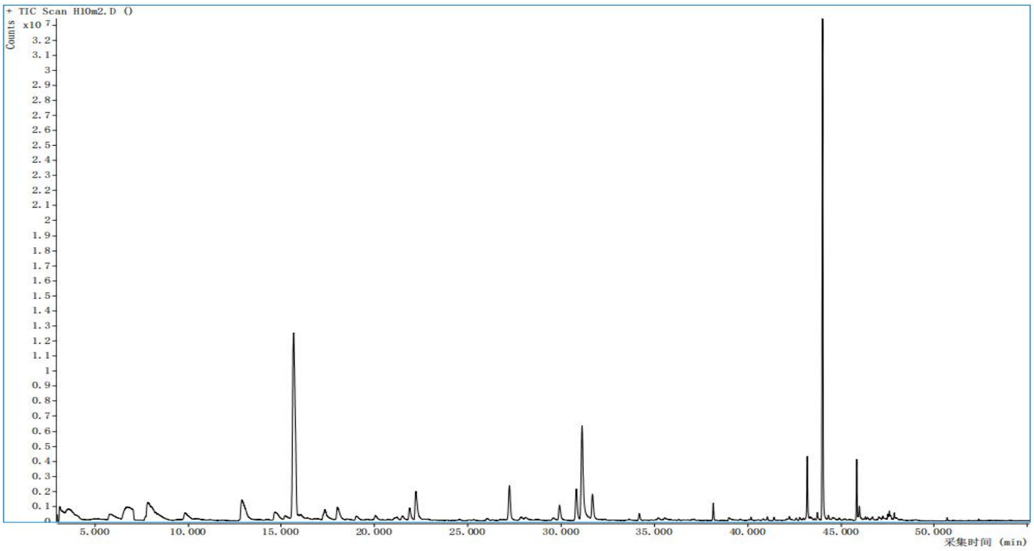
+ TIC Scan H10m2.D ()
Counts
x10 7
采集时间 (min)

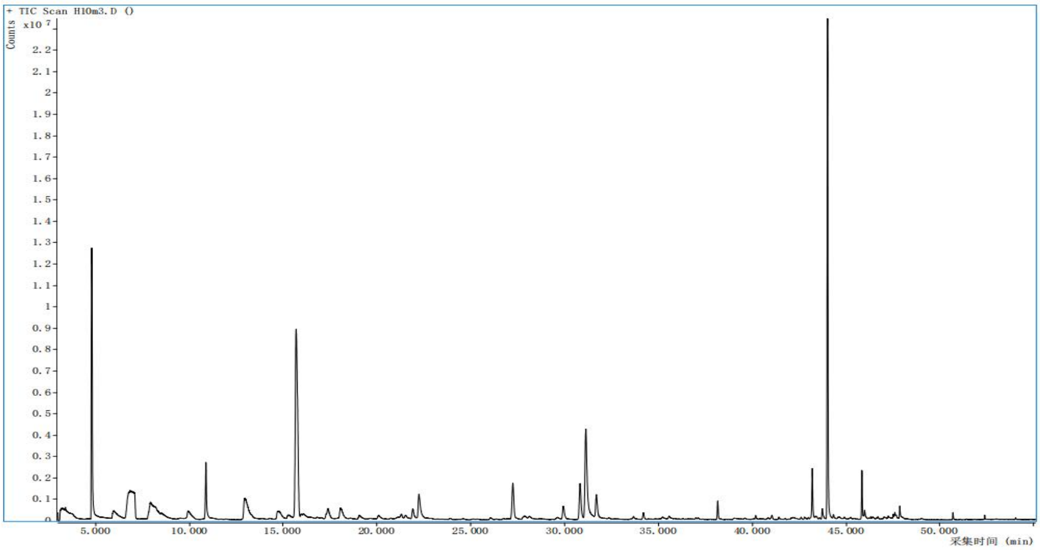
+ TIC Scan H10m3.D ()
Counts
x10 7
采集时间 (min)

(H) H30m

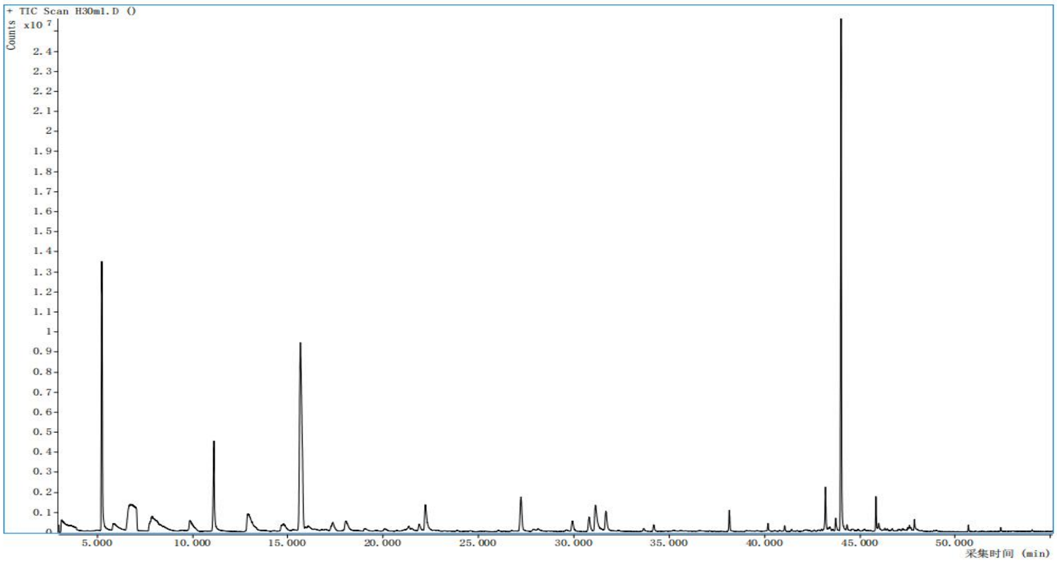

+ TIC Scan H30m1.D ()
Counts
x10 7
采集时间 (min)

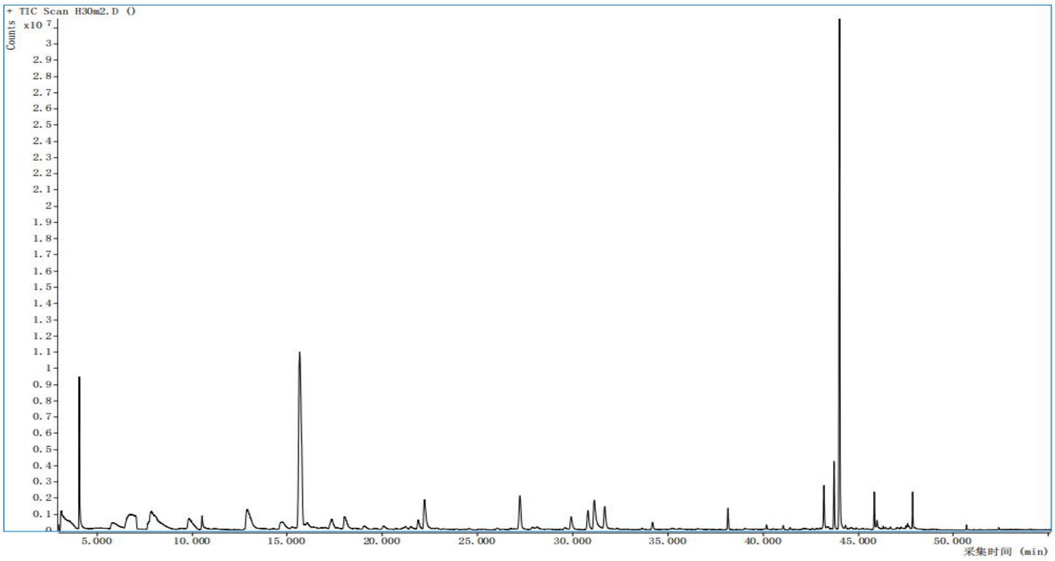

+ TIC Scan H30m2.D ()
Counts
x10 7
采集时间 (min)

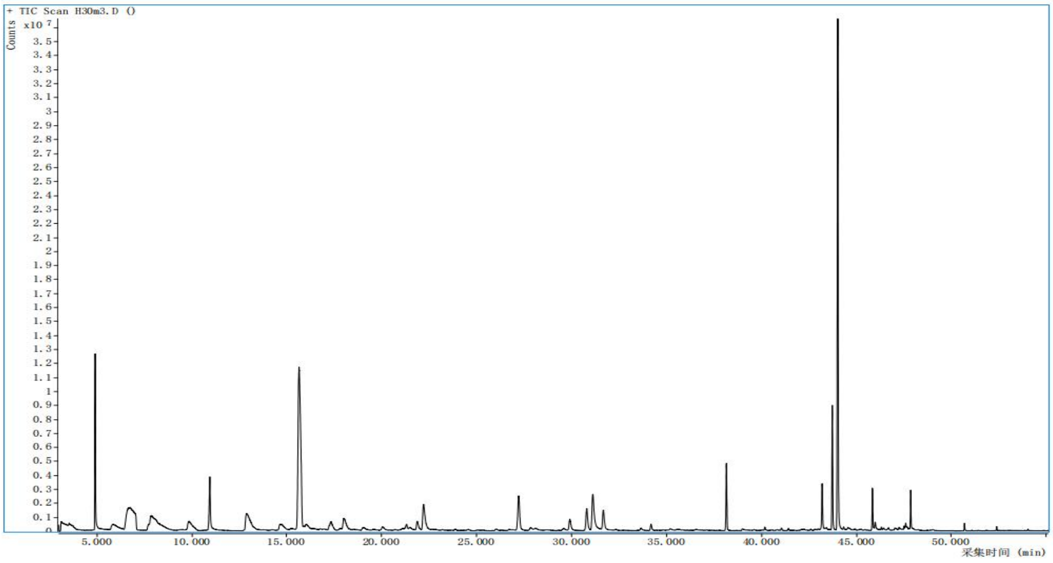

+ TIC Scan H30m3.D ()
Counts
x10 7
采集时间 (min)

(I) H1h

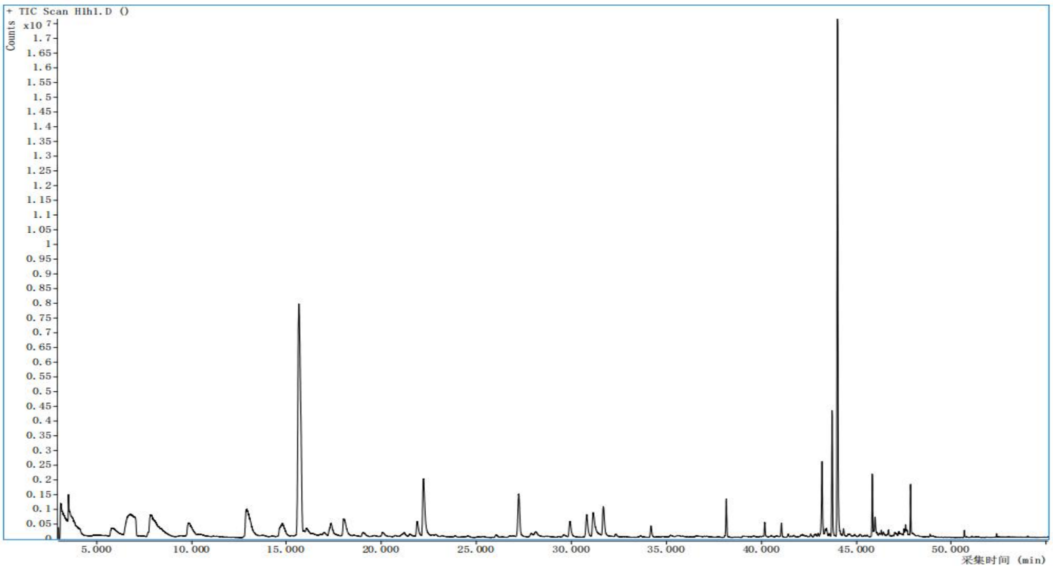
+ TIC Scan H1h1.D ()
Counts
x10 7
5.000
10.000
15.000
20.000
25.000
30.000
35.000
40.000
45.000
50.000
采集时间 (min)

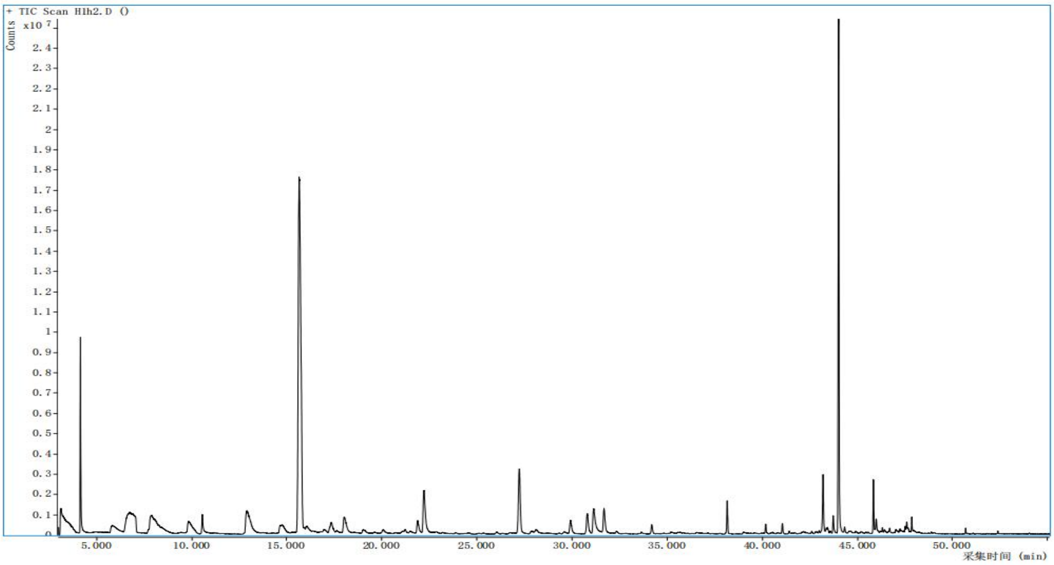
+ TIC Scan H1h2.D ()
Counts
x10 7
5.000
10.000
15.000
20.000
25.000
30.000
35.000
40.000
45.000
50.000
采集时间 (min)

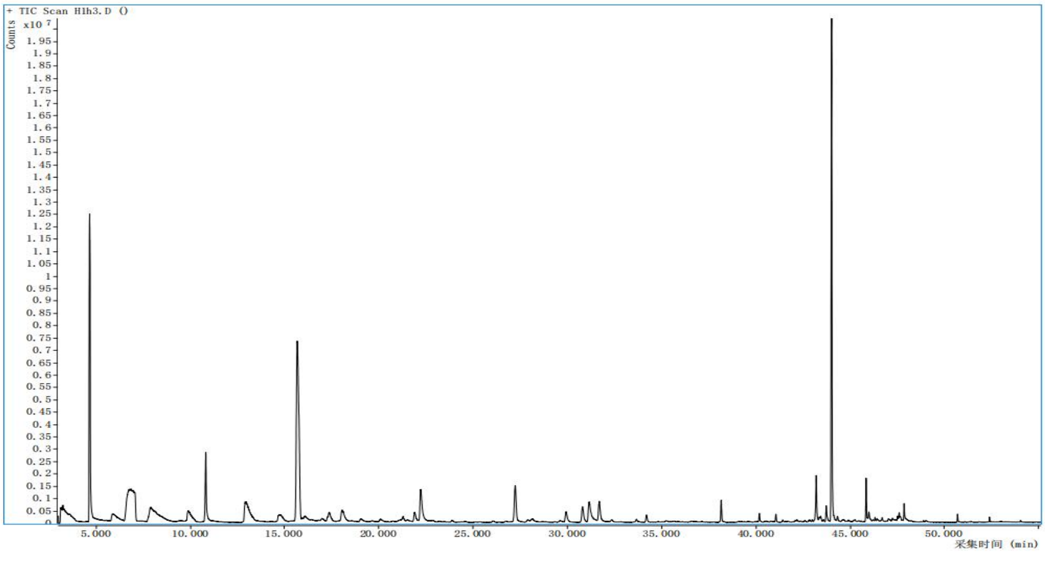
+ TIC Scan H1h3.D ()
Counts
x10 7
5.000
10.000
15.000
20.000
25.000
30.000
35.000
40.000
45.000
50.000
采集时间 (min)

(J) H2h

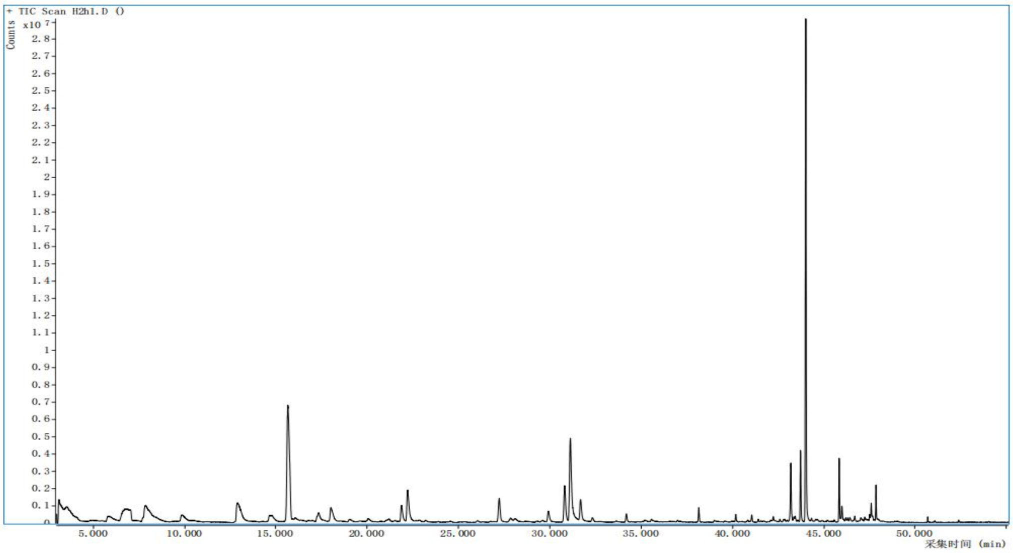

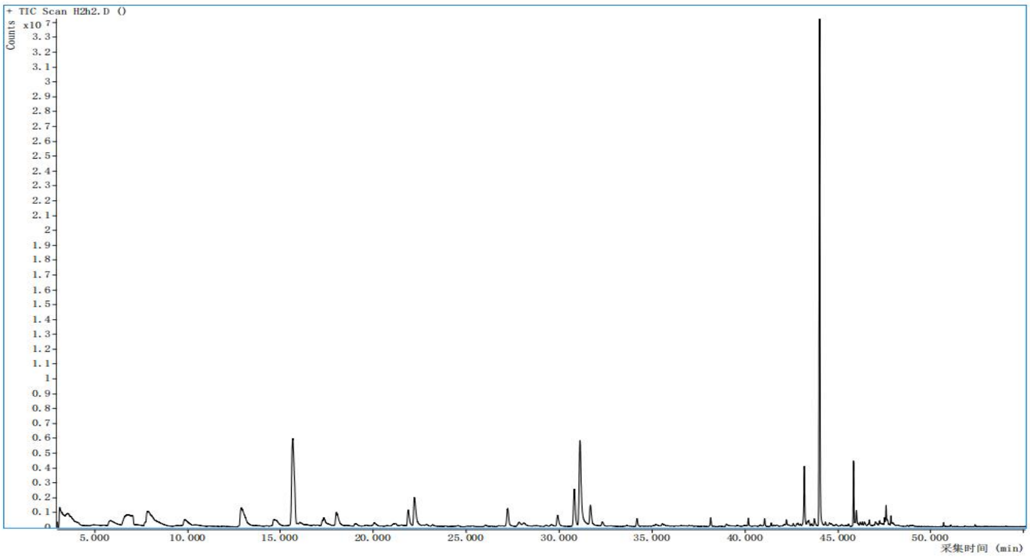

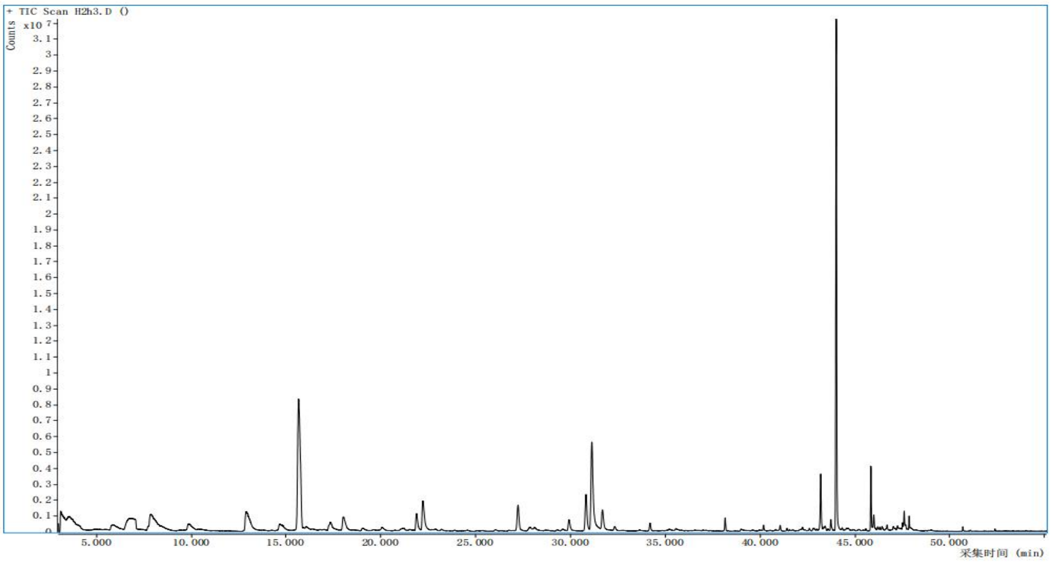

Figure S1. Total ion chromatograms (TICs) obtained by HS-SPME-GC-MS analysis of cold-

and hot-brewed *C. paliurus* tea infusions. Note: Total ion chromatograms of three independently prepared replicates are shown for each brewing treatment: (A) C5m; (B) C10m; (C) C30m; (D) C1h; (E) C2h; (F) H5m; (G) H10m; (H) H30m; (I) H1h; and (J) H2h. C and H indicate cold brewing (4 °C) and hot brewing (90 °C), respectively; 5m, 10m, 30m, 1h, and 2h indicate brewing durations of 5 min, 10 min, 30 min, 1 h, and 2 h, respectively. The chromatograms were obtained from *C. paliurus* tea infusions analyzed by headspace solid-phase microextraction coupled with gas chromatography–mass spectrometry (HS-SPME-GC-MS). The x-axis represents retention time (min), and the y-axis represents ion intensity. Volatile compounds were tentatively annotated based on mass spectral matching against the NIST 23 library and retention-index (RI) consistency, with candidate annotations retained when $|\Delta RI| \leq 30$. Because authentic reference standards were not analyzed, all compound assignments should be regarded as tentative. Corresponding retention times, experimental RIs, reference RIs, relative contents, and RSD values are provided in Supplementary Table S5.

Figure S2. Quality-control evaluation of the untargeted metabolomics dataset

A：Hotelling’s T2 plot

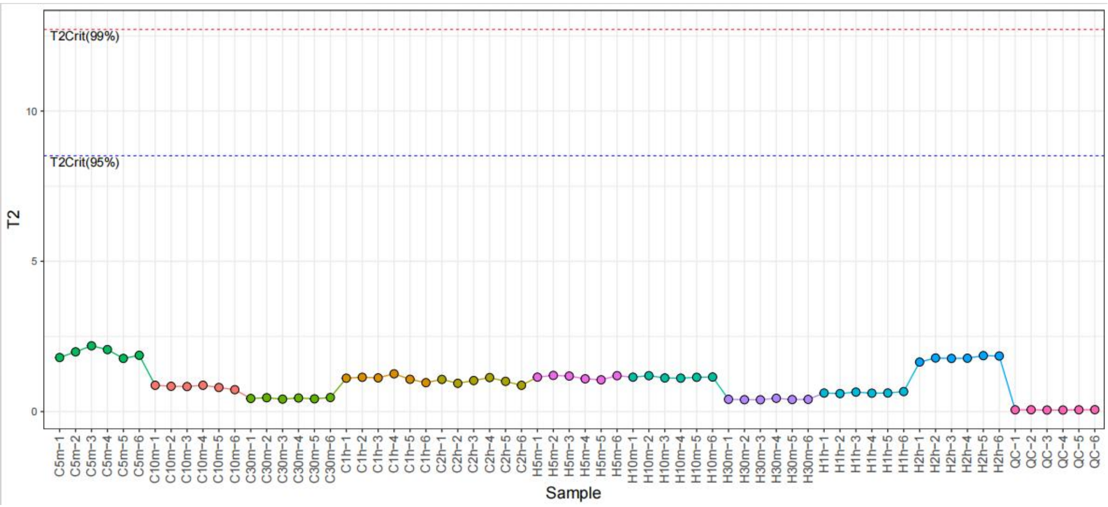

B：PCA score plot including QC samples

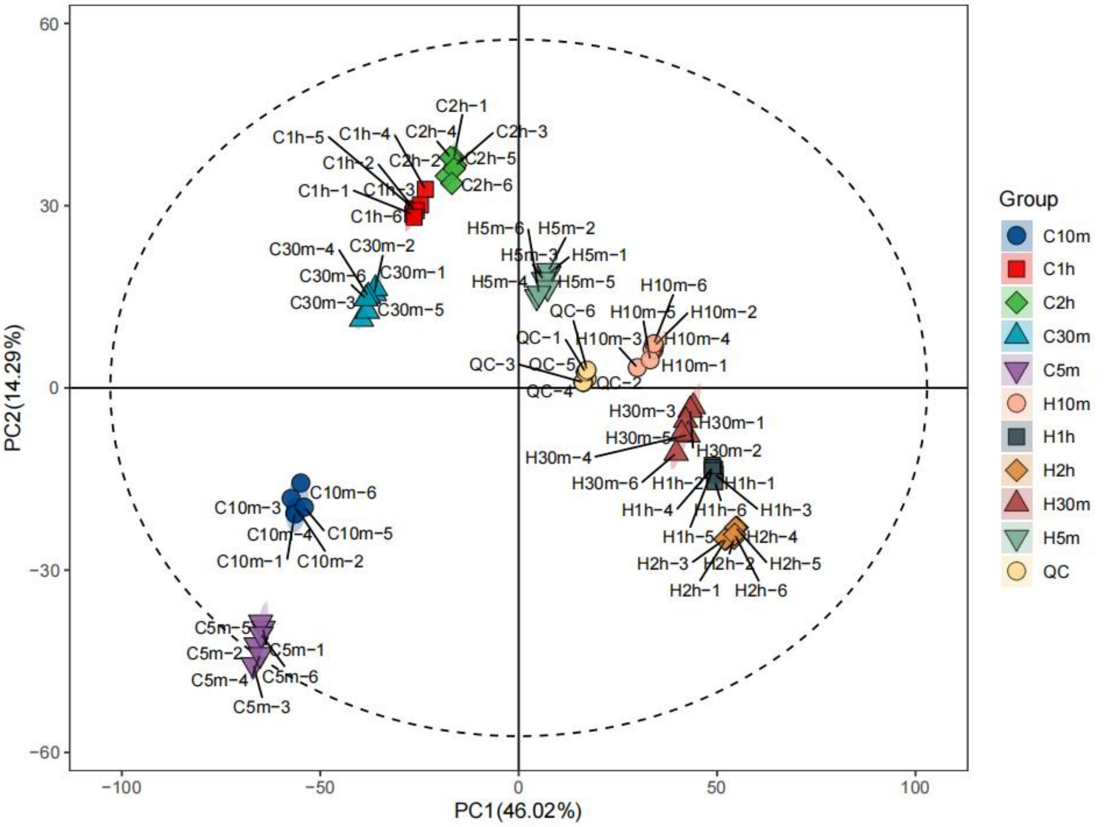

Figure S2. Quality-control assessment of the untargeted metabolomics dataset. Notes: (A) Hotelling’s T2 plot of the untargeted metabolomics dataset. The dashed horizontal lines indicate the 95% and 99% confidence limits, and the overall sample distribution remained within the predefined confidence boundaries. (B) PCA score plot of experimental and QC samples based on the overall metabolite profiles. The close clustering of QC samples supported satisfactory analytical stability and reproducibility of the UHPLC-HRMS/MS platform.

Figure S3. Temperature- and time-dependent variation in the number of detected volatile features in cold- and hot-brewed *C. paliurus* tea infusions.

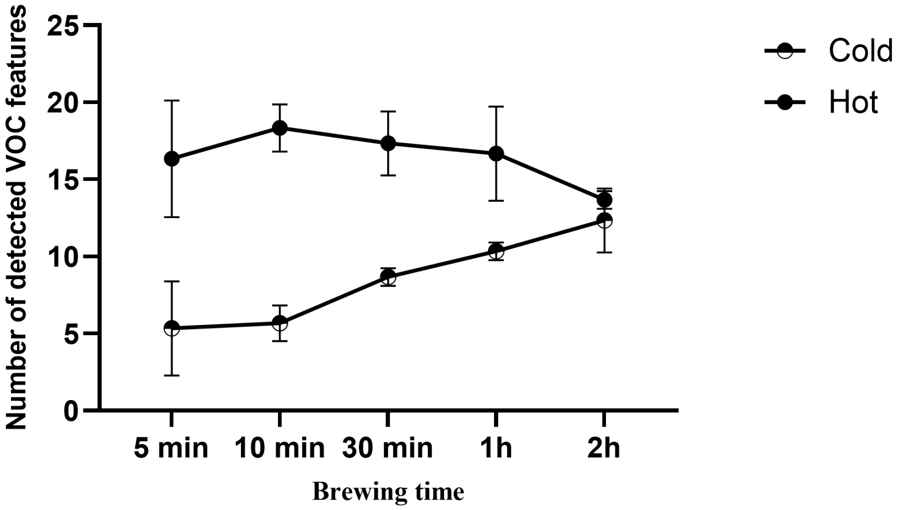

Figure S3. Temperature- and time-dependent variation in the number of detected volatile features in cold- and hot-brewed *C. paliurus* tea infusions. The number of detected features was calculated for each independently prepared sample from the 61 tentatively annotated volatile features retained in the HS-SPME-GC-MS dataset. Values are presented as mean ± SD (n = 3). Cold and hot brewing were performed at 4 ℃ and 90 ℃, respectively. A significant temperature × time interaction was observed using a Poisson generalized linear model with a log link function (Wald $\chi^2 = 12.641$, df = 4, $p = 0.013$), supported by ordinary two-way ANOVA ($F(4, 20) = 6.349$, $p = 0.0018$). Feature counts reflect detection within the retained feature set rather than the total number of chromatographic peaks.

Table

Table S1. Panelist-to-panelist variability in sensory evaluation of cold- and hot-brewed *C. paliurus* tea infusions.

Sensory attribute	Panelist F(8,72)	*p* value	Median r	Median CV (%)
Aroma	10.595	<0.001	0.611	49.1
Sweetness	2.243	0.0338	0.773	52.5
Bitterness	7.292	<0.001	0.945	49.6
Sourness	7.005	<0.001	0.924	49.5
Umami	6.988	<0.001	0.507	52.8
Thickness	12.290	<0.001	0.890	34.5

Notes: Nine panelists (P1–P9) each evaluated all ten treatments once (one score per panelist × treatment). F and p values refer to panelist effects in exploratory two-way additive ANOVA (treatment and panelist as factors), without independent replicate observations per cell; the panelist × treatment interaction cannot be estimated separately. Median r is the median Pearson correlation between each panelist's ten-treatment score profile and the corresponding panel-mean profile. Median CV is the median across-treatment coefficient of variation among the nine panelist scores (sample SD / mean × 100%). These analyses describe panelist variability and are not evidence of independent infusion replicates. Individual scores and detailed calculations are retained in the accompanying verified Excel workbook.

Table S2. Electronic tongue response values of cold- and hot-brewed *C. paliurus* tea infusions under different brewing conditions.

Taste attribute	Tasteless point	C5m	C10m	C30m	C1h	C2h	H5m	H10m	H30m	H1h	H2h
Sourness	-13	-26.22 ± 0.00	-25.69 ± 0.15	-25.30 ± 0.26	-24.90 ± 0.26	-25.13 ± 0.25	-23.12 ± 0.26	-22.65 ± 0.17	-22.17 ± 0.10	-21.71 ± 0.07	-21.46 ± 0.03
Bitterness	0	1.66 ± 0.00	3.52 ± 0.08	4.13 ± 0.06	7.85 ± 0.04	9.51 ± 0.10	15.88 ± 0.16	18.46 ± 0.23	18.42 ± 0.26	19.28 ± 0.31	19.59 ± 0.37
Astringency	0	-3.41 ± 0.00	-1.44 ± 0.22	-1.97 ± 0.10	1.96 ± 0.16	4.25 ± 0.16	8.27 ± 0.23	9.99 ± 0.20	10.70 ± 0.16	10.80 ± 0.11	10.90 ± 0.07
Aftertaste-B	0	0.57 ± 0.00	1.00 ± 0.02	1.24 ± 0.01	1.85 ± 0.02	2.07 ± 0.02	4.58 ± 0.06	5.89 ± 0.09	6.09 ± 0.07	6.59 ± 0.13	6.81 ± 0.09
Aftertaste-A	0	0.62 ± 0.00	0.80 ± 0.03	0.91 ± 0.02	1.31 ± 0.03	1.52 ± 0.03	2.85 ± 0.01	3.21 ± 0.02	3.45 ± 0.01	3.46 ± 0.04	3.44 ± 0.03
Umami	0	9.71 ± 0.00	10.16 ± 0.11	10.32 ± 0.06	10.33 ± 0.02	10.25 ± 0.04	10.04 ± 0.06	9.97 ± 0.04	9.84 ± 0.05	9.60 ± 0.09	9.51 ± 0.09
Richness	0	0.75 ± 0.00	0.81 ± 0.15	1.03 ± 0.06	1.13 ± 0.05	1.06 ± 0.03	1.75 ± 0.03	2.12 ± 0.04	2.37 ± 0.03	2.35 ± 0.04	2.42 ± 0.02
Saltiness	-6	-13.53 ± 0.00	-12.12 ± 0.23	-10.82 ± 0.10	-9.99 ± 0.09	-10.44 ± 0.12	-7.47 ± 0.10	-6.62 ± 0.11	-5.73 ± 0.06	-5.93 ± 0.06	-5.69 ± 0.05
Sweetness	0	12.91 ± 0.00	11.43 ± 0.02	8.75 ± 0.01	9.36 ± 0.06	7.94 ± 0.02	8.13 ± 0.02	7.62 ± 0.05	7.40 ± 0.04	7.40 ± 0.04	7.13 ± 0.02

Note: Values are presented as mean ± SD (n = 3 independent brewing replicates). C and H indicate cold brewing (4 ℃) and hot brewing (90 ℃), respectively; 5m, 10m, 30m, 1h, and 2h indicate brewing durations of 5 min, 10 min, 30 min, 1 h, and 2 h, respectively. The tasteless points for the sourness and saltiness sensors were established at −13 and −6, respectively, whereas a uniform tasteless point of 0 was applied across all remaining taste attributes. . Aftertaste-B and Aftertaste-A represent bitter aftertaste and astringent aftertaste, respectively. Richness represents the persistence of umami (umami aftertaste).

Table S3. Contents of bioactive components in cold- and hot-brewed *C. paliurus* tea infusions.

Sample	TPC (mg/g)	TFC (mg/g)	TP (mg/g)	TSC (mg/g)
C5m	23.90 ± 6.12	0.0052 ± 0.0007	7.46 ± 5.77	0.00 ± 0.00
C10m	36.51 ± 4.13	0.0082 ± 0.0004	20.39 ± 6.68	0.00 ± 0.00
C30m	68.06 ± 6.69	0.0130 ± 0.0006	51.63 ± 4.55	0.00 ± 0.00
C1h	84.76 ± 15.14	0.0178 ± 0.0030	81.95 ± 5.06	0.00 ± 0.00
C2h	100.78 ± 5.83	0.0235 ± 0.0008	93.37 ± 7.85	0.00 ± 0.00
H5m	120.32 ± 6.55	0.0149 ± 0.0027	43.54 ± 10.08	9.16 ± 0.65
H10m	197.54 ± 6.75	0.0423 ± 0.0004	69.80 ± 8.34	15.52 ± 0.12
H30m	233.07 ± 18.72	0.0600 ± 0.0020	84.21 ± 5.87	17.85 ± 0.69
H1h	269.12 ± 24.58	0.0708 ± 0.0039	115.22 ± 10.17	18.84 ± 0.83
H2h	289.83 ± 3.02	0.0771 ± 0.0004	171.04 ± 12.83	20.89 ± 0.51

Notes: Values are expressed as mean ± SD (n = 3 independent brewing replicates). TPC, total phenolic content; TFC, total flavonoid content; TP, total polysaccharide content; TSC, total saponin content.

Table S4. DPPH and ABTS radical-scavenging capacities of cold- and hot-brewed *C. paliurus* tea infusions.

Treatment	DPPH radical-scavenging capacity (%)	ABTS radical-scavenging capacity (%)
C5m	18.14 ± 1.33	18.73 ± 0.55
C10m	26.73 ± 2.03	24.13 ± 0.66
C30m	30.91 ± 2.22	30.64 ± 0.70
C1h	67.79 ± 0.98	39.02 ± 1.03
C2h	47.93 ± 1.84	36.24 ± 1.25
H5m	49.84 ± 0.64	49.73 ± 0.98
H10m	60.62 ± 0.93	53.17 ± 2.71
H30m	70.50 ± 2.59	61.15 ± 3.57
H1h	87.78 ± 1.29	71.81 ± 1.54
H2h	90.84 ± 0.51	85.68 ± 2.86

Notes: Values are expressed as mean ± SD (n = 3 independent brewing replicates). DPPH, 2,2-diphenyl-1-picrylhydrazyl; ABTS, 2,2′-azino-bis(3-ethylbenzothiazoline-6-sulfonic acid). Radical-scavenging capacity is expressed as percentage (%).

Table S5. Volatile compounds tentatively annotated in cold- and hot-brewed *C. paliurus* tea infusions by HS-SPME-GC-MS.
(A) Cold-brewed tea infusions — C5m - C2h

Class	Compound name	CAS No.	Mean RT(min)	C5m				C10m				C30m				C1h				C2h			
				Relative content(%)	Calc. RI	Ref. RI	RSD (%)	Relative content(%)	Calc. RI	Ref. RI	RSD (%)	Relative content(%)	Calc. RI	Ref. RI	RSD (%)	Relative content(%)	Calc. RI	Ref. RI	RSD (%)	Relative content(%)	Calc. RI	Ref. RI	RSD (%)
Aldehydes	(1R,4aR,5S)-5-[(E)-5-Hydroxy-3-methylpent-3-enyl]-1,4a-dimethyl-6-methylidene-3,4,5,7,8,8a-hexahydro-2H-naphthalene-1-carbaldehyde	1214984-94-7	46.00																				
	(E)-Hexadec-2-enal	22644-96-8	36.99																				
	1-Cyclohexene-1-carboxaldehyde	1192-88-7	16.10																				
	2,4-Heptadien-6-ynal, (E,E)-	7200-04-6	12.92																	10.73	983.30	999	28.02
	2,4-Heptadienal, (E,E)-	4313-03-5	15.24																				
	Benzaldehyde	100-52-7	12.93	15.02	985.87	962	0.86					26.94	983.14	962	13.75	23.54	983.47	962	3.41	19.93	983.03	962	5.85
	Butanal, 2-methyl-	96-17-3	3.12	30.06	662.87	662	29.31	31.13	662.95	662	14.32	25.23	662.55	662	18.01	23.60	663.12	662	29.37	19.93	662.76	662	29.87
	Heptanal	111-71-7	9.86																	3.59	903.98	901	6.51
	Pentanal, 3-methyl-	15877-57-3	5.86																				
Alcohols/Phenols	1-(1H-Imidazol-2-yl)-2,2-dimethyl-propan-1-one	61985-30-6	25.38																				
	2,4-Dimethylcyclopentanol	89794-28-5	9.87																				
	2-Naphthalenol, acetate	1523-11-1	31.77																				

	2-Nonen-1-ol, 2-methyl-	91008-40-1	22.22													9.16	1233.31	1234	10.27				
	3-Hexen-1-ol, (Z)-	928-96-1	7.88																				
	4-Hexen-1-ol	6126-50-7	7.92					24.52	848.70	848	28.60	28.31	846.59	848	14.14	19.24	847.22	848	30.07	15.00	849.40	848	28.54
	5-(2,5-Dimethoxy-phenyl)-2H-pyrazol-3-ol	1000278-26-9	42.60													0.54	2238.94	2240	5.84	0.78	2238.93	2240	12.69
	6-Chromanol	5614-78-8	29.92													5.78	1509.10	1509	1.20	6.07	1509.66	1509	11.12
	6-Octen-1-ol, 3,7-dimethyl-, acetate	150-84-5	26.03																				
	8-Decen-1-ol, 5,9-dimethyl-	69303-22-6	27.78																				
	Bicyclo[3.1.1]heptan-3-ol, 2,6,6-trimethyl-	473-61-0	20.09													3.14	1172.78	1173	14.21				
	Bicyclo[4.1.0]heptan-3-ol, 3,7,7-trimethyl-, [1S-(1.alpha.,3.beta.,6.alpha.)]-	4017-92-9	21.49									3.00	1212.43	1212	0.27	2.56	1211.53	1212	5.58	2.44	1211.99	1212	33.20
	E,Z-8,10-Dodecadien-1-ol	1000130-96-3	30.82																	5.03	1551.48	1550	30.99
	Isomaltol	3420-59-5	14.64																	1.65	1017.61	989	47.40
	p-Cumenol	99-89-8	21.90									0.89	1223.79	1224	25.10								
Ketones	3,6,6-TRIMETHYL-CYCLOHEX-2-ENONE	23438-77-9	19.03	1.70	1143.88	1152	26.87	3.16	1145.03	1152	35.13	3.55	1143.52	1152	17.09	3.40	1143.62	1152	2.20	3.06	1143.65	1152	8.74
	3-Octen-2-one, 7-methyl-	33046-81-0	17.34																				
	3-Pentanone	96-22-0	4.03																				
	Cyclopropyl 2-thienyl ketone	6193-47-1	26.03																				
	Elsholtzia ketone	488-05-1	21.13																				
Esters	3-Hydroxy-4-isopropylphenyl, acetate	1010548-14-0	31.71	3.19	1592.88	1596	29.42																
	3-Isopropylphenyl N-methylcarbamate (isomer 2)	1000470-98-9	32.35																				

	Benzoic acid, 2-(1-methylpropyl)oxy-, methyl ester	1000375-33-4	29.56																				
	Fenchyl acetate	13851-11-1	21.88									4.91	1223.47	1224	7.39	4.68	1223.75	1224	9.83	4.63	1223.45	1224	0.29
	l-Alanine, N-(4-butylbenzoyl)-, pentyl ester	1000314-15-4	47.31																				
	Phthalic acid, hex-3-yl undecyl ester	1000356-96-2	51.25	1.16	2751.62	2754	34.74																
	Propanoic acid, ethenyl ester	105-38-4	3.50																				
	Vinyl crotonate	14861-06-4	7.87																				
Hydrocarbons	.beta.-Phellandrene	555-10-2	14.79																	4.13	1031.79	1031	33.08
	1,3-Cyclopentadiene, 5-(1-methylethylidene)-	2175-91-9	8.28																				
	2,4-Hexadiene	592-46-1	3.11																				
	Benzene, 1,4-dimethoxy-	150-78-7	18.56																				
	Cyclohexene, 1-methyl-4-(1-methylethylidene)-	586-62-9	16.97																				
	Cyclopentane, decyl-	1795-21-7	30.84									3.67	1552.18	1554	2.61								
	Cyclopropane, octyl-	1472-09-9	17.25	45.57	1095.16	1095	11.08	23.87	1096.29	1095	2.04												
	Hexadecane, 2,6,10,14-tetramethyl-	638-36-8	35.67	0.78	1801.09	1802	31.89																
	Oxirane, heptadecyl-	67860-04-2	40.84																				
	Oxirane, octyl-	2404-44-6	22.22					14.41	1233.90	1230	22.99												
	Propane, 2-isocyanato-	1795-48-8	3.14																				
Nitrogen	1H-Pyrazole, 1,3-dimethyl-	694-48-4	8.13																				
	3-(Methylaminomethyl)phenylamine, 2Ac derivative	1458490-01-1	42.60	2.52	2239.21	2239	14.53	0.80	2238.87	2239	1.71	0.48	2239.17	2239	29.73								

comp	3-Pentanamine	616-24-0	3.19																
ounds	4-(3-Acetyl-phenoxy)-phthalonitrile	313249-32-0	46.53																
	Cyclohexylamine	108-91-8	8.45									1.74	862.77	863	12.34				
	Propionamide, 3-(3,3-dimethyl-3,4-dihydro-2H-isoquinolin-1-ylidene)-2-oxo-	1000304-68-5	44.90													0.35	2387.88	2388	32.03
	2-Propenoic acid	79-10-7	5.88																
	3-Acetyl-2,5-dimethyl furan	10599-70-9	18.56																
	4-Amino-N-ethylphthalimide	55080-55-2	39.24																
Other s	4-Oxo-4-(5,6,7,8-tetrahydro-2-naphthalenyl)butanoic acid	785-17-1	42.09																
	cis-Decalin, 2-syn-methyl-	1000155-85-6	20.06																
	Empenthrin	54406-48-3	34.19	2.12	1720.80	1721	14.39	3.01	1720.37	1721	4.31	2.63	1720.14	1721	7.44	2.69	1720.59	1721	14.08
	N-(p-Vinylbenzoyl)-l-alanine	139184-60-4	39.60																

(B) Hot-brewed tea infusions — H5m - H2h

Class	Compound name	CAS No.	Mean RT(min)	H5m				H10m				H30m				H1h				H2h			
				Relative content(%)	Calc. RI	Ref. RI	RSD (%)	Relative content(%)	Calc. RI	Ref. RI	RSD (%)	Relative content(%)	Calc. RI	Ref. RI	RSD (%)	Relative content(%)	Calc. RI	Ref. RI	RSD (%)	Relative content(%)	Calc. RI	Ref. RI	RSD (%)
Aldehydes	(1R,4aR,5S)-5-[(E)-5-Hydroxy-3-methylpent-3-enyl]-1,4a-dimethyl-6-methylidene-3,4,5,7,8,8a-hexahydro-2H-naphthalene-1-carbaldehyde	1214984-94-7	46.00									2.02	2443.96	2441	29.40								
	(E)-Hexadec-2-enal	22644-96-8	36.99																	0.59	1877.70	1878	30.55
	1-Cyclohexene-1-carboxaldehyde	1192-88-7	16.10									4.30	1055.59	1029	4.36								
	2,4-Heptadien-6-ynal, (E,E)-	7200-04-6	12.92									20.24	982.66	999	18.73								
	2,4-Heptadienal, (E,E)-	4313-03-5	15.24	2.66	1041.28	1012	34.79																
	Benzaldehyde	100-52-7	12.93	18.54	982.63	962	21.85	19.67	982.22	962	21.98					23.32	982.89	962	16.96	24.61	982.58	962	12.12
	Butanal, 2-methyl-	96-17-3	3.12	20.56	663.57	662	29.17					10.14	663.92	662	36.80								
	Heptanal	111-71-7	9.86	5.94	903.85	901	30.05					7.74	903.63	901	23.95	12.73	903.86	901	10.65	8.41	903.14	901	2.38
	Pentanal, 3-methyl-	15877-57-3	5.86	6.00	779.47	781	4.80	5.64	780.68	781	35.24	5.90	780.80	781	21.02	6.77	782.43	781	5.38	7.19	780.18	781	24.75
Alcohols/Phenols	1-(1H-Imidazol-2-yl)-2,2-dimethyl-propan-1-one	61985-30-6	25.38													0.22	1331.28	1329	28.45				
	2,4-Dimethylcyclopentanol	89794-28-5	9.87					4.76	904.09	906	0.19												
	2-Naphthalenol, acetate	1523-11-1	31.77									0.06	1595.74	1596	12.15								

	2-Nonen-1-ol, 2-methyl-	91008-40-1	22.22																				
	3-Hexen-1-ol, (Z)-	928-96-1	7.88					21.29	846.04	857	12.89												
	4-Hexen-1-ol	6126-50-7	7.92	14.28	846.68	848	26.27					19.70	845.86	848	10.95	18.94	846.54	848	30.07				
	5-(2,5-Dimethoxy-phenyl)-2H-pyrazol-3-ol	1000278-26-9	42.60	0.30	2238.96	2240	29.59	0.44	2238.38	2240	26.07	0.23	2238.94	2240	22.90	0.58	2238.68	2240	24.50	0.80	2238.69	2240	6.20
	6-Chromanol	5614-78-8	29.92	6.54	1509.08	1509	0.24	5.94	1509.10	1509	24.53	5.14	1509.01	1509	17.57	5.32	1509.68	1509	19.68	5.78	1509.34	1509	8.00
	6-Octen-1-ol, 3,7-dimethyl-, acetate	150-84-5	26.03	1.28	1354.45	1354	2.10																
	8-Decen-1-ol, 5,9-dimethyl-	69303-22-6	27.78					0.87	1419.52	1420	34.02									0.89	1419.58	1420	16.27
	Bicyclo[3.1.1]heptan-3-ol, 2,6,6-trimethyl-	473-61-0	20.09																				
	Bicyclo[4.1.0]heptan-3-ol, 3,7,7-trimethyl-, [1S-(1.alpha.,3.beta.,6.alpha.)]-	4017-92-9	21.49	1.99	1212.20	1212	10.57	1.76	1211.50	1212	37.45	1.52	1211.99	1212	35.90								
	E,Z-8,10-Dodecadien-1-ol	1000130-96-3	30.82																				
	Isomaltol	3420-59-5	14.64																				
	p-Cumenol	99-89-8	21.90																				
Ketones	3,6,6-TRIMETHYL-CYCLOHEX-2-ENONE	23438-77-9	19.03	2.04	1143.50	1152	24.84	2.27	1143.69	1152	34.34	1.92	1144.83	1152	31.34	1.95	1143.49	1152	12.74	2.33	1143.56	1152	16.33
	3-Octen-2-one, 7-methyl-	33046-81-0	17.34					7.12	1098.23	1098	21.26	6.84	1097.98	1098	16.58	6.63	1098.09	1098	17.59				
	3-Pentanone	96-22-0	4.03	2.74	702.37	688	38.36																
	Cyclopropyl 2-thienyl ketone	6193-47-1	26.03													0.24	1354.47	1355	18.87				
	Elsholtzia ketone	488-05-1	21.13	1.41	1201.28	1202	32.32																
Esters	3-Hydroxy-4-isopropylphenyl, acetate	1010548-14-0	31.71																				
	3-Isopropylphenyl N-methylcarbamate (isomer 2)	1000470-98-9	32.35									0.61	1624.29	1624	35.67	0.91	1625.71	1624	20.32	2.12	1624.03	1624	7.26

	Benzoic acid, 2-(1-methylpropyl)oxy-, methyl ester	1000375-33-4	29.56	0.52	1493.45	1496	5.75																
	Fenchyl acetate	13851-11-1	21.88	3.66	1223.06	1224	35.92	3.89	1223.12	1224	27.89	4.01	1223.35	1224	32.62	4.69	1223.34	1224	20.37	8.15	1223.31	1224	4.28
	l-Alanine, N-(4-butylbenzoyl)-, pentyl ester	1000314-15-4	47.31					0.13	2511.70	2509	22.51												
	Phthalic acid, hex-3-yl undecyl ester	1000356-96-2	51.25																				
	Propanoic acid, ethenyl ester	105-38-4	3.50													4.21	679.61	668	34.45				
	Vinyl crotonate	14861-06-4	7.87					4.18	846.00	817	31.68												
Hydrocarbons	.beta.-Phellandrene	555-10-2	14.79	4.77	1031.44	1031	15.87	3.20	1031.54	1031	1.61	3.06	1031.47	1031	36.24	4.68	1031.70	1031	35.79				
	1,3-Cyclopentadiene, 5-(1-methylethylidene)-	2175-91-9	8.28	0.57	858.00	858	2.95																
	2,4-Hexadiene	592-46-1	3.11																	19.64	662.67	663	21.05
	Benzene, 1,4-dimethoxy-	150-78-7	18.56													0.27	1139.92	1168	28.62	0.23	1139.40	1168	23.09
	Cyclohexene, 1-methyl-4-(1-methylethylidene)-	586-62-9	16.97									0.51	1088.40	1088	14.47	2.14	1088.16	1088	24.54				
	Cyclopentane, decyl-	1795-21-7	30.84																				
	Cyclopropane, octyl-	1472-09-9	17.25																				
	Hexadecane, 2,6,10,14-tetramethyl-	638-36-8	35.67																				
	Oxirane, heptadecyl-	67860-04-2	40.84					0.38	2119.78	2115	32.34									0.42	2119.16	2115	6.01
	Oxirane, octyl-	2404-44-6	22.22					10.71	1232.78	1230	34.26									15.27	1232.79	1230	4.94
	Propane, 2-isocyanato-	1795-48-8	3.14									2.10	663.95	663	14.46								
Nitrogen	1H-Pyrazole, 1,3-dimethyl-	694-48-4	8.13					2.52	852.77	854	5.22					3.04	853.97	854	28.15				
	3-(Methylaminomethyl)phenylamine, 2Ac derivative	1458490-01-1	42.60																				

compounds	3-Pentanamine	616-24-0	3.19	2.84	666.08	692	31.49																
	4-(3-Acetyl-phenoxy)-phthalonitrile	313249-32-0	46.53													0.04	2470.92	2474	1.02				
	Cyclohexylamine	108-91-8	8.45																				
	Propionamide, 3-(3,3-dimethyl-3,4-dihydro-2H-isoquinolin-1-ylidene)-2-oxo-	1000304-68-5	44.90	0.43	2387.51	2388	27.70	0.45	2387.45	2388	29.59	0.35	2387.43	2388	16.27	0.47	2387.14	2388	34.46	0.43	2387.73	2388	17.79
Others	2-Propenoic acid	79-10-7	5.88									1.24	781.62	784	35.27								
	3-Acetyl-2,5-dimethyl furan	10599-70-9	18.56	0.94	1071.01	1095	21.04																
	4-Amino-N-ethylphthalimide	55080-55-2	39.24					0.04	2016.02	2020	33.77												
	4-Oxo-4-(5,6,7,8-tetrahydro-2-naphthalenyl)butanoic acid	785-17-1	42.09					0.10	2203.41	2206	25.10												
	cis-Decalin, 2-syn-methyl-	1000155-85-6	20.06					2.21	1172.06	1172	30.58												
	Empenthrin	54406-48-3	34.19	2.00	1719.48	1721	26.11	2.29	1719.67	1721	18.09	2.39	1719.88	1721	16.52	2.87	1720.10	1721	19.61	3.15	1719.90	1721	6.82
	N-(p-Vinylbenzoyl)-l-alanine	139184-60-4	39.60					0.13	2039.05	2041	26.01												

Note: C and H indicate cold brewing (4 °C) and hot brewing (90 °C), respectively; 5m, 10m, 30m, 1h, and 2h indicate the corresponding brewing durations. RT, retention time; Calc. RI, experimentally calculated retention index; Ref. RI, reference retention index; RSD, relative standard deviation. Relative content (%) was calculated from the mean peak area of each retained compound relative to the total mean peak area within the corresponding treatment, and RSD (%) was calculated from the valid replicate peak-area measurements (n = 2 or 3). Volatile compounds were tentatively annotated by mass spectral matching and RI consistency, with candidate annotations retained when $|\Delta RI| \leq 30$; blank cells indicate compounds not retained under the applied screening criteria.

Table S6. Classification, tentative annotation, and statistical information of the 50 core differential metabolite features selected from cold- and hot-brewed *C. paliurus* tea infusions.

Superclass	Metabolite	Chemical class	Formula	VIP	p-value	FDR	Brewing response
Benzenoids	m-Carboxyphenyl phenylacetamidomethylphosphonate	Benzene and substituted derivatives	C16H16NO6P	1.13856	1.8E-11	3.07E-10	Cold-enriched
	1'-Acetoxyeugenol acetate	Phenol esters	C14H16O5	1.05288	8.82883E-05	0.000185588	Cold-enriched
Lipids and lipid-like molecules	Physalin Y	Steroids and steroid derivatives	C28H32O10	1.65411	2.05872E-06	6.14098E-06	Hot-enriched
	5alpha,6beta,8alpha-Trihydroxy-28-norisotoonafolin	Prenol lipids	C25H30O9	1.41514	7.271E-09	4.3697E-08	Hot-enriched
	2-aminohexadecanoic acid	Fatty Acyls	C16H33NO2	1.37536	1E-12	2.8E-11	Cold-enriched
	Parthenolide	Prenol lipids	C15H20O3	1.33035	2.06E-10	2.123E-09	Cold-enriched
	Platanic acid	Steroids and steroid derivatives	C29H46O4	1.21343	2.3339E-08	1.09274E-07	Hot-enriched
	Hymenoside R	Fatty Acyls	C19H26O10	1.13723	1.38296E-07	5.15762E-07	Cold-enriched
	Notoginsenoside R2	Prenol lipids	C41H70O13	1.12602	2.30518E-07	8.44237E-07	Cold-enriched
	LysoPA(18:2(9Z,12Z)/0:0)	Glycerophospholipids	C21H39O7P	1.08992	1.3045E-08	6.5872E-08	Hot-enriched

Sargaol	Prenol lipids	C27H38O2	1.0751	1E-12	2.2E-11	Cold-enriched
Dianoside A	Prenol lipids	C42H66O15	1.05326	9.63E-10	7.247E-09	Cold-enriched
Docosanedioic acid	Fatty Acyls	C22H42O4	1.0397	4.65566E-05	0.000100221	Hot-enriched
Taxuside	Fatty Acyls	C16H24O8	1.03462	2.6374E-08	1.17005E-07	Cold-enriched
Deacylgymnemic acid	Prenol lipids	C36H58O12	1.03185	3.1907E-08	1.43699E-07	Cold-enriched
Hippuristerone K	Steroids and steroid derivatives	C33H52O7	1.03143	4.43679E-05	9.82202E-05	Hot-enriched
Triflorestevione	Prenol lipids	C25H36O6	1.02799	0	0	Hot-enriched
Ebracteatoside D	Fatty Acyls	C19H34O11	1.0159	1.59E-10	1.471E-09	Cold-enriched
Auraptene	Prenol lipids	C19H22O3	1.01492	1.0114E-06	2.91833E-06	Cold-enriched
LysoPC(18:1/0:0)	Glycerophospholipids	C26H52NO7P	1.01453	1.53E-10	1.694E-09	Hot-enriched
2-Methoxyoctadecanoic acid	Fatty Acyls	C19H38O3	1.01241	3.3471E-05	6.85119E-05	Cold-enriched
(13S,14S)-epoxymaresin	Fatty Acyls	C22H30O3	1.00754	0	2E-12	Cold-enriched
Caryocaroside II-5	Prenol lipids	C36H56O10	1.00708	0	5E-12	Hot-enriched
beta-fenchocamphorone	Prenol lipids	C11H18O	1.00676	1.429E-09	1.0015E-08	Cold-enriched

	(2R,3R,4S,5R,6R)-2-[[7-[(2R,3R,4R,5S)-3,4-dihydroxy-5-(hydroxymethyl)oxolan-2-yl]oxy-2-ethenyl-2,4b,8,8-tetramethyl-4,4a,5,6,7,8a,9,10-octahydro-3H-phenanthren-3-yl]oxy]-6-methyloxane-3,4,5-triol	Prenol lipids	C31H50O10	1.00625	0.000356529	0.000652865	Cold-enriched
	Sericoside	Prenol lipids	C36H58O11	1.00483	0	2.3E-11	Hot-enriched
Organic acids and derivatives	Hypaphorine	Carboxylic acids and derivatives	C14H18N2O2	1.03994	0	1E-12	Hot-enriched
	N-Lauroylglycine	Carboxylic acids and derivatives	C14H27NO3	1.01392	2.9E-11	4.68E-10	Cold-enriched
	Myristoylglycine	Carboxylic acids and derivatives	C16H31NO3	1.00596	1.983E-09	1.3338E-08	Cold-enriched
Organic nitrogen compounds	Sphingosine	Organonitrogen compounds	C18H37NO2	1.26756	2E-12	6.9E-11	Cold-enriched
Organoheterocyclic compounds	Caffeine	Imidazopyrimidines	C8H10N4O2	1.66356	0	1E-12	Hot-enriched
	Stolonic acid B	Dioxanes	C26H44O5	1.34204	5.34E-10	4.653E-09	Cold-enriched
	Lancerin	Benzopyrans	C19H18O10	1.22754	1.676E-09	1.1979E-08	Hot-enriched
	alpha-Mangostin	Benzopyrans	C24H26O6	1.14599	8E-12	1.53E-10	Hot-enriched

	Methyl pheophorbide a	Tetrapyrroles and derivatives	C36H38N4O5	1.12923	2.65218E-06	7.59129E-06	Cold-enriched
	12-(3,4-diethoxyphenyl)-2,3-diethoxy-10-(prop-2-yn-1-yloxy)-5,6-dihydroindolo[2,1-a]isoquinoline	Pyrroles	C33H35NO5	1.07535	3.61E-10	2.918E-09	Hot-enriched
	Ascorbigen	Furofurans	C15H15NO6	1.04223	7.3554E-07	2.22666E-06	Hot-enriched
	Mukoenine B	Indoles and derivatives	C23H25NO2	1.02833	0	1E-12	Hot-enriched
	Epoxypheophorbide a	Tetrapyrroles and derivatives	C35H36N4O6	1.02476	2.687E-09	1.704E-08	Hot-enriched
Phenylpropanoids and polyketides	Wedelolactone	Isoflavonoids	C16H10O7	1.20021	0	0	Hot-enriched
	Ferulagol A	Coumarins and derivatives	C19H22O4	1.09053	1.1E-11	2.03E-10	Hot-enriched
	Kaempferol 3-O-arabinoside	Flavonoids	C20H18O10	1.08189	0	2E-12	Hot-enriched
	8-C-p-Hydroxybenzylkaempferol	Diarylheptanoids	C22H16O7	1.07685	2E-12	5.7E-11	Hot-enriched
	Gossypetin 8-glucuronide	Flavonoids	C21H18O14	1.06038	2.71505E-07	1.00281E-06	Hot-enriched
	5-hydroxy-7-(2-(4-(2-hydroxyethyl)piperazin-1-yl)-2-oxoethoxy)-2-	Flavonoids	C23H24N2O6	1.04623	4.45966E-07	1.58171E-06	Hot-enriched

	phenyl-4H-chromen-4-one						
	Zeranol	Macrolides and analogues	C18H26O5	1.04547	6E-12	1.03E-10	Cold-enriched
	Lunarine	Macrolactams	C25H31N3O4	1.03751	1.718E-09	1.1299E-08	Hot-enriched
	5-Hydroxy-3,6,7,8,3',4',5'-heptamethoxyflavone	Flavonoids	C22H24O10	1.00755	8.35728E-07	2.48482E-06	Cold-enriched
	Irisflorentin	Isoflavonoids	C20H18O8	1.0051	2.62786E-06	7.71315E-06	Cold-enriched
	Broussoflavan A	Flavonoids	C25H30O6	1.00361	1.7299E-08	8.605E-08	Cold-enriched

Note: The 50 metabolites correspond to those displayed in Fig. 4E. Metabolites were classified according to their chemical superclass and chemical class. VIP values were obtained from the OPLS-DA model. “Cold-enriched” and “Hot-enriched” indicate relatively higher abundance under cold- and hot-brewing conditions, respectively.

Table S7. Two-way ANOVA and HC3 robust interaction analysis of 50 representative differential metabolite features in cold- and hot-brewed *C. paliurus* tea infusions

Alignment ID	Tentative annotation	Temperature	Time	Interaction	HC3 interaction
		F / q	F / q	F / q	F / q
NEG5048	m-Carboxyphenyl phenylacetamidomethylphosphonate	88.8 / 2.00e-12	58.9 / 1.33e-17	135 / 5.24e-25	122 / 3.90e-24
NEG3100	1'-Acetoxyeugenol acetate	38.9 / 1.38e-07	11.7 / 1.55e-06	5.57 / 0.00135	2.38 / 0.0781
POS9911	Physalin Y	1.58 / 0.231	6.76 / 0.00029	38.8 / 4.06e-14	32.7 / 7.85e-13
NEG7353	5alpha,6beta,8alpha-Trihydroxy-28-norisotoonafolin	202 / 8.37e-19	134 / 4.98e-25	35 / 2.59e-13	44 / 3.81e-15
POS4484	2-aminohexadecanoic acid	589 / 1.10e-28	94 / 9.95e-22	54.7 / 6.76e-17	44 / 3.90e-15
POS3401	Parthenolide	71.3 / 5.69e-11	41.3 / 1.06e-14	154 / 2.76e-26	189 / 3.00e-28
NEG7072	Platanic acid	144 / 5.22e-16	6.41 / 0.000435	2.13 / 0.108	1.6 / 0.212
NEG6268	Hymenoside R	281 / 1.00e-21	18 / 7.29e-09	5.34 / 0.00177	4.26 / 0.00673
POS11706	Notoginsenoside R2	463 / 2.35e-26	44.3 / 3.07e-15	109 / 5.14e-23	128 / 1.62e-24
NEG6673	LysoPA(18:2(9Z,12Z)/0:0)	387 / 1.15e-24	21.2 / 6.68e-10	76.1 / 9.51e-20	57.2 / 2.66e-17
POS7505	Sargaol	376 / 2.18e-24	63.2 / 3.33e-18	316 / 3.79e-33	302 / 1.03e-32
NEG10355	Dianoside A	116 / 2.55e-14	14.4 / 1.33e-07	132 / 8.22e-25	125 / 2.54e-24
NEG5489	Docosanedioic acid	61.5 / 4.64e-10	2.44 / 0.0688	24.4 / 1.00e-10	22 / 4.76e-10
POS6370	Taxuside	587 / 1.19e-28	83.2 / 1.25e-20	5.53 / 0.00141	3.47 / 0.0186
NEG9591	Deacylgymnemic acid	55.9 / 1.70e-09	10.7 / 4.30e-06	55.5 / 5.13e-17	62.8 / 4.15e-18
NEG8531	Hippuristerone K	17.1 / 0.000171	3.88 / 0.0102	22.5 / 3.35e-10	15.1 / 8.62e-08
NEG6637	Triflorestevione	3e+03 / 4.61e-45	43.7 / 3.86e-15	14.4 / 1.56e-07	15.6 / 5.63e-08
NEG6744	Ebracteatoside D	1.18e+03 / 1.28e-35	74.1 / 1.35e-19	37.7 / 6.92e-14	32.4 / 9.22e-13
NEG3847	Auraptene	189 / 3.02e-18	36.1 / 1.18e-13	27.2 / 1.76e-11	22.6 / 3.18e-10
NEG8596	LysoPC(18:1/0:0)	469 / 1.70e-26	5.27 / 0.00175	15.1 / 8.73e-08	13.4 / 3.75e-07
NEG4263	2-Methoxyoctadecanoic acid	87.5 / 2.53e-12	7.3 / 0.000156	6.88 / 0.000288	10.1 / 8.46e-06

POS6331	(13S,14S)-epoxymaresin	955 / 1.76e-33	94 / 9.98e-22	270 / 1.22e-31	276 / 7.28e-32
POS10910	Caryocaroside II-5	361 / 5.03e-24	20.5 / 1.11e-09	7.14 / 0.000213	47.3 / 1.00e-15
POS1742	beta-fenchocamphorone	632 / 2.21e-29	62.9 / 3.64e-18	49.2 / 5.12e-16	55.1 / 5.36e-17
NEG8719	(2R,3R,4S,5R,6R)-2-[[7-[(2R,3R,4R,5S)-3,4-dihydroxy-5-(hydroxymethyl)oxolan-2-yl]oxy-2-ethenyl-2,4b,8,8-tetramethyl-4,4a,5,6,7,8a,9,10-octahydro-3H-phenanthren-3-yl]oxy]-6-methyloxane-3,4,5-triol	45.9 / 2.04e-08	6.03 / 0.000684	20.6 / 1.29e-09	18.3 / 6.78e-09
NEG9448	Sericoside	523 / 1.56e-27	55.5 / 4.22e-17	6.87 / 0.000289	11.2 / 2.91e-06
POS3844	Hypaphorine	1.07e+04 / 5.48e-58	146 / 8.78e-26	236 / 2.52e-30	244 / 1.06e-30
NEG2928	N-Lauroylglycine	139 / 9.39e-16	28 / 9.08e-12	17.6 / 1.17e-08	18.3 / 6.69e-09
NEG3540	Myristoylglycine	96.6 / 5.14e-13	25.8 / 3.38e-11	15.1 / 9.20e-08	11.1 / 3.30e-06
POS5218	Sphingosine	215 / 2.31e-19	27.5 / 1.23e-11	18.3 / 6.79e-09	12.7 / 6.89e-07
POS2423	Caffeine	2.04e+03 / 3.90e-41	7.25e+03 / 7.38e-65	5.37e+03 / 1.31e-61	6.54e+03 / 9.80e-64
NEG6719	Stolonic acid B	4.94 / 0.0352	26.4 / 2.36e-11	42.4 / 8.09e-15	58.8 / 1.51e-17
POS8183	Lancerin	434 / 9.45e-26	55.6 / 4.09e-17	75.5 / 1.12e-19	68.9 / 6.57e-19
NEG6214	alpha-Mangostin	520 / 1.79e-27	76.5 / 7.18e-20	26.6 / 2.55e-11	26.1 / 3.39e-11
POS10583	Methyl pheophorbide a	89.8 / 1.68e-12	17.2 / 1.30e-08	113 / 2.49e-23	128 / 1.62e-24
POS10119	12-(3,4-diethoxyphenyl)-2,3-diethoxy-10-(prop-2-yn-1-yloxy)-5,6-dihydroindolo[2,1-a]isoquinoline	489 / 6.92e-27	17.1 / 1.40e-08	7.62 / 0.000126	67.3 / 1.06e-18
POS5373	Ascorbigen	434 / 9.49e-26	10.9 / 3.53e-06	7.37 / 0.000166	5.92 / 0.000869
POS6462	Mukoenine B	2.33e+03 / 1.74e-42	34.6 / 2.50e-13	61.5 / 6.77e-18	154 / 2.58e-26
POS10598	Epoxypheophorbide a	11.6 / 0.00161	2.33 / 0.08	1.06 / 0.41	0.783 / 0.564
POS5581	Wedelolactone	2.27e+03 / 3.03e-42	590 / 3.23e-39	2.8e+03 / 4.80e-55	2.23e+03 / 1.79e-52
NEG4252	Ferulagol A	2.23e+03 / 4.53e-42	83.4 / 1.19e-20	90.8 / 2.37e-21	61.4 / 6.52e-18
NEG6345	Kaempferol 3-O-arabinoside	1.26e+04 / 1.18e-59	226 / 5.97e-30	344 / 6.11e-34	286 / 3.47e-32
NEG5902	8-C-p-Hydroxybenzylkaempferol	1.97e+03 / 8.95e-41	139 / 2.20e-25	140 / 2.19e-25	107 / 6.71e-23
NEG7659	Gossypetin 8-glucuronide	178 / 9.69e-18	36.9 / 8.23e-14	6.9 / 0.00028	9.11 / 2.43e-05
POS8106	5-hydroxy-7-(2-(4-(2-hydroxyethyl)piperazin-1-yl)-2-oxoethoxy)-2-phenyl-4H-chromen-4-one	130 / 3.05e-15	2.25 / 0.0879	3.2 / 0.0267	5.9 / 0.000889
NEG4429	Zeranol	1.13e+03 / 3.93e-35	230 / 3.76e-30	126 / 2.18e-24	83.5 / 1.27e-20

POS8354	Lunarine	452 / 4.01e-26	12.4 / 7.81e-07	11 / 3.71e-06	7.82 / 9.73e-05
NEG6915	5-Hydroxy-3,6,7,8,3',4',5'-heptamethoxyflavone	318 / 7.37e-23	17.9 / 7.89e-09	7.84 / 9.76e-05	8.6 / 4.16e-05
POS7317	Irisflorentin	211 / 3.63e-19	12.1 / 1.08e-06	6.91 / 0.000276	6.62 / 0.00038
NEG6510	Broussoflavan A	231 / 5.85e-20	26.7 / 1.93e-11	2.4 / 0.0761	2.21 / 0.0975

Note: Values are reported as F statistic / BH-FDR-adjusted q-value. Temperature: $F(1,50)$; time and temperature × time interaction: $F(4,50)$. BH correction was applied separately for each effect across all 3,666 annotated features. HC3 denotes a heteroscedasticity-consistent joint F-test for the interaction. $q < 0.05$ was considered significant. Feature names are tentative database annotations; $n = 6$ independent samples per treatment. The unabridged statistics, including raw p-values and diagnostics, are retained in the master Excel file.
